# Structural basis for receptor-regulated SMAD recognition by MAN1

**DOI:** 10.1093/nar/gky925

**Published:** 2018-10-13

**Authors:** Ken-ichi Miyazono, Yosuke Ohno, Hikaru Wada, Tomoko Ito, Yui Fukatsu, Akira Kurisaki, Makoto Asashima, Masaru Tanokura

**Affiliations:** 1 Department of Applied Biological Chemistry, Graduate School of Agricultural and Life Sciences, The University of Tokyo, Tokyo 113-8657, Japan; 2 Graduate School of Biological Sciences, Nara Institute of Science and Technology, Nara, Japan; 3 Biotechnology Research Institute for Drug Discovery (BRD), National Institute of Advanced Industrial Science and Technology (AIST), Ibaraki, Japan

## Abstract

Receptor-regulated SMAD (R-SMAD: SMAD1, SMAD2, SMAD3, SMAD5 and SMAD8) proteins are key transcription factors of the transforming growth factor-β (TGF-β) superfamily of cytokines. MAN1, an integral protein of the inner nuclear membrane, is a SMAD cofactor that terminates TGF-β superfamily signals. Heterozygous loss-of-function mutations in *MAN1* result in osteopoikilosis, Buschke-Ollendorff syndrome and melorheostosis. MAN1 interacts with MAD homology 2 (MH2) domains of R-SMAD proteins using its C-terminal U2AF homology motif (UHM) domain and UHM ligand motif (ULM) and facilitates R-SMAD dephosphorylation. Here, we report the structural basis for R-SMAD recognition by MAN1. The SMAD2–MAN1 and SMAD1–MAN1 complex structures show that an intramolecular UHM–ULM interaction of MAN1 forms a hydrophobic surface that interacts with a hydrophobic surface among the H2 helix, the strands β8 and β9, and the L3 loop of the MH2 domains of R-SMAD proteins. The complex structures also show the mechanism by which SMAD cofactors distinguish R-SMAD proteins that possess a highly conserved molecular surface.

## INTRODUCTION

The transforming growth factor-β (TGF-β) superfamily proteins, including TGF-β, Nodal, Activin and BMP, are multifunctional cytokines and control various processes that are involved in embryonic development and adult tissue homeostasis (1–3). Due to their multifunctionality, the dysregulation of TGF-β superfamily signaling causes various disease, such as cancer and fibrosis (4, 5). TGF-β superfamily signals are transduced into the cell through heteromeric serine/threonine kinase receptor complexes (TGF-β type I receptors (TβRI) and type II receptors (TβRII)). Once the TGF-β superfamily signals are recognized by the receptor complexes, the receptors phosphorylate the conserved C-terminal SXS (Ser-X-Ser) motif of the receptor-regulated SMAD (R-SMAD: SMAD1, SMAD 2, SMAD 3, SMAD 5 and SMAD 8) proteins (Supplementary Figure S1A). The TGF-β, Nodal and Activin (TGF-β/Nodal/Activin) signals stimulate phosphorylation of SMAD2 and SMAD3, whereas the BMP signals stimulate phosphorylation of SMAD1, SMAD5 and SMAD8. Two molecules of phosphorylated R-SMAD proteins form heteromeric complexes with one common-mediator SMAD (Co-SMAD: SMAD4) protein and translocate into the nucleus to regulate the expression of many signal-dependent genes (6). The activated receptor complexes also stimulate other signaling pathways to regulate TGF-β-dependent biological processes (7).

R-SMAD proteins are key regulators of the TGF-β superfamily signaling pathway in cells. R-SMAD proteins are transcription factors that possess an N-terminal MAD homology 1 (MH1) domain that is used for DNA binding and a C-terminal MH2 domain that is used for protein-protein interactions. These two domains are connected by a poorly conserved disordered linker segment (Supplementary Figure S2A, B) (6). In most cases, R-SMAD proteins function cooperatively with other proteins (SMAD cofactors) (8). The multifunctionality of the TGF-β superfamily signals is largely due to the diversity of SMAD cofactors. The BioGRID database (version 3.4.159) (9) shows that SMAD2 and SMAD3 from humans interact with 264 and 347 proteins, respectively. By contrast, SMAD1, SMAD5 and SMAD8 from humans interact with 122, 62 and 114 proteins, respectively. Among these R-SMAD-binding proteins, 97 proteins interact with both groups of R-SMAD proteins and regulate both TGF-β/Nodal/Activin and BMP signals. Many SMAD cofactors bind to the MH2 domains of R-SMAD proteins. For example, the membrane-associated SMAD anchor for receptor activation (SARA) recruits SMAD2 and SMAD3, whereas the membrane-associated endosome-associated FYVE domain protein (ENDOFIN) recruits SMAD1, SMAD5 and SMAD8 to the TGF-β receptor complexes to facilitate R-SMAD phosphorylation (10–13). In the nucleus, R-SMAD proteins form complexes with transcriptional coactivators (for example, p300 and CREB binding protein (CBP)) (14), transcriptional corepressors (e.g. proto-oncoprotein SKI, SKI-related protein (SNON) and TGF-β-induced factor homeobox (TGIF)) (15–18), and transcription factors (for example, Forkhead box protein H1 (FOXH1), Mix-like endodermal regulator (Mixer) and SMAD-interacting protein 1 (SIP1)) (19–21) to activate or repress gene expression. The MH2 domains of R-SMAD proteins are highly conserved. The MH2 domains of SMAD2 and SMAD3 and those of SMAD1, SMAD5, and SMAD8 share 97% and 90% amino acid sequence identity, respectively. Meanwhile, the MH2 domains of all R-SMAD proteins share 74% amino acid sequence identity. Each SMAD cofactor that binds to the MH2 domains can recognize these amino acid sequence differences to bind to SMAD2 and SMAD3 or SMAD1, SMAD5 and SMAD8, or both.

MAN antigen 1 (MAN1) is a one of the SMAD cofactors that represses TGF-β superfamily protein signals (22, 23). MAN1 is an integral inner nuclear membrane protein that contains two transmembrane helices in its middle region. In the N-terminal region, MAN1 possesses an LEM (LAP2, emerin, MAN1) domain that mediates protein-protein interactions (24). In the C-terminal region, MAN1 possesses a winged helix (WH) DNA-binding domain and a U2AF homology motif (UHM) domain (Supplementary Figure S2C) (25,26). These domains are exposed to the nucleoplasm. A UHM domain is a non-canonical RNA-recognition motif (RRM) that mediates protein-protein interactions with a protein that contains a U2AF ligand motif (ULM) (27). At the inner nuclear membrane, MAN1 directly interacts with the MH2 domains of R-SMAD proteins using the UHM domain and facilitates R-SMAD dephosphorylation (22,23). Loss-of-function mutations in *MAN1*, which result in loss of the R-SMAD binding domain of MAN1, cause osteopoikilosis, Buschke-Ollendorff syndrome and melorheostosis. These phenotypes can be explained by the enhanced TGF-β superfamily signal (28). The R-SMAD binding by MAN1 requires highly conserved tryptophan and glutamine residues in a ULM sequence that lies between the WH domain and the UHM domain (Supplementary Figure S2C) (26). Mutation assays and small-angle X-ray scattering (SAXS) assays have shown that SMAD2 binds to MAN1 near the H2 helix (Tyr366 and Trp368) of the SMAD2 MH2 domain (29). However, Tyr366 and Trp368 of SMAD2 are not conserved in SMAD1, SMAD5 and SMAD8 (Supplementary Figure S1A). The precise structural basis for R-SMAD recognition by MAN1, especially the mechanism by which the UHM domain and ULM of MAN1 are used for R-SMAD recognition, remains unclear.

To clarify the R-SMAD binding mechanism by MAN1, we determined the structures of the SMAD2–MAN1 and SMAD1–MAN1 complexes by X-ray crystallography. Based on the structures and the accompanying biochemical data, we have revealed the structural basis for the R-SMAD recognition mechanism by the intramolecular UHM–ULM complex of MAN1; the hydrophobic surface of MAN1 that is stabilized by the intramolecular UHM–ULM interaction binds to the conserved hydrophobic surface of R-SMAD proteins. A structural comparison with other SMAD-cofactor complexes shows the mechanism by which SMAD cofactors select their binding targets.

## MATERIALS AND METHODS

### Protein expression and purification

Gene fragments of human *SMAD2* (NM_005901) and *MAN1* (NM_001167614) were amplified by PCR from cDNA and were cloned into the pET-48b (+) plasmid (Novagen) (pET48-SMAD2C-dC, pET48-SMAD2C-2E, pET48-MAN1(762–890) and pET48-MAN1(762–911)). A gene fragment of human *SMAD2* and *SMAD1* (NM_005900) were amplified by PCR from cDNA and were cloned into the pGEX-6P-3 plasmid (GE Healthcare, pGEX-6P-SMAD2C-2E and pGEX-6P-SMAD1C-2E). The protein constructs of SMAD2, SMAD1 and MAN1 used in this study are summarized in Supplementary Figure S2. Each plasmid was modified by the PrimeSTAR Mutagenesis Basal Kit (TAKARA). For the co-purification of SMAD2C-dC with MAN1(762–890), the 6 × histidine tag (His-tag) region of pET48-SMAD2C-dC was removed (pET48-SMAD2C-dC-w/o-His). For the His-tag pull-down assay, the pGEX-6P-SMAD2C-2E and pGEX-6P-SMAD1C-2E plasmids were modified to express N-terminal His- and glutathione S-transferase (GST)-tagged SMAD2C-2E and SMAD1C-2E (His-GST-SMAD2C-2E and His-GST-SMAD1C-2E). The pET48-MAN1(762–890) and pET48-MAN1(762–911) plasmids were modified to express N-His-tag-HRV3C protease site-Trx tag-Gly-Ser-MAN1-Ser-Asp-Glu-Asp-C (His-Trx-MAN1(762–890)-SDED and His-Trx-MAN1(762–911)-SDED). The pET48-SMAD2C-2E was also modified to express N-His-tag-HRV3C protease site-Trx tag-Gly-Ser-SMAD2C-2E-C (His-Trx-SMAD2C-2E). The gene fragments of human *SKI* (NM_003036) and CBP (NM_004380) were also cloned into the pET-48b (+) plasmid and modified to express N-His-tag-HRV3C protease site-Trx tag-Gly-Ser-SKI (residues 16–40)-Ser-Asp-Glu-Asp-C (His-Trx-SKI(16–40)-SDED) and N-His-tag-HRV3C protease site-Trx tag-Gly-Ser-CBP (residues 1941–1973)-Ser-Asp-Glu-Asp-C (His-Trx-CBP(1941-1973)-SDED).

The expression plasmids of the W765A, F770A, R775A, W855A and L860A mutants of MAN1 were prepared by modifying the expression plasmids of the His-Trx-MAN1(762–890)-SDED and His-Trx-MAN1(762–911)-SDED using the PrimeSTAR Mutagenesis Basal Kit (TAKARA). The expression plasmids of the Y366A, W368A, P377A and Y366H mutants and the Y366H-W368F double mutant of SMAD2 were prepared by modifying the His-Trx-SMAD2C-2E expression plasmid using the same method. The expression plasmid of the H364Y-F366W double mutant of SMAD1 was prepared by modifying the His-GST-SMAD1C-2E expression plasmid using the same method.

For SMAD2 and SMAD1 expression, the constructed plasmids were transformed into *E. coli* BL21(DE3) cells (Novagen) harboring the pG-KJE8 plasmid (TAKARA). The transformants were cultivated at 37°C in LB medium supplemented with 20 μg/ml kanamycin or 50 μg/ml ampicillin, 50 μg/ml chloramphenicol, 0.5 mg/ml arabinose and 5 ng/ml tetracycline until the optical density at 600 nm reached 0.6. For MAN1, SKI and CBP expression, the constructed plasmids were transformed into *Escherichia coli* Rosetta (DE3) cells (Novagen). The transformants were cultivated at 37°C in LB medium supplemented with 20 μg/ml kanamycin and 50 μg/ml chloramphenicol until the optical density at 600 nm reached 0.6. The expression of each protein was induced by the addition of isopropyl β-d-thiogalactopyranoside (IPTG) at a final concentration of 0.1 mM (SMAD2, SMAD1, MAN1 and CBP) or 1 mM (SKI). After cultivation at 25°C (SMAD2, SMAD1, MAN1 and SKI) or 18°C (CBP) overnight, the cells were harvested by centrifugation at 5,000 × g for 10 min.

For cocrystallization of the SMAD2–MAN1 and SMAD1–MAN1 complexes, *E. coli* cells overexpressing SMAD2 (pET48-SMAD2C-dC-w/o-His) or SMAD1 (pGEX-6P-SMAD1-2E), and MAN1 (pET48-MAN1(762–890)) were mixed and resuspended in 50 mM Tris–HCl (pH 9.0), 200 mM NaCl, 10 mM imidazole, 10% glycerol and 1 mM Tris(2-carboxyethyl)phosphine (TCEP). The cells were lysed by sonication and were centrifuged at 40 000 × g for 30 min. The supernatants were purified using Ni-NTA Superflow resin (QIAGEN). The complexes were eluted with a buffer solution containing 50 mM Tris–HCl (pH 9.0), 200 mM NaCl, 200 mM imidazole, 10% glycerol and 1 mM TCEP. The eluted solutions were treated by HRV3C protease to cleave the fused Trx- and GST-tags (4°C, overnight). The treated solutions were concentrated by ammonium sulfate precipitation (40% saturation) and were purified using a Superdex 200 HR 10/30 column (GE Healthcare) that was pre-equilibrated with buffer containing 10 mM Tris–HCl (pH 9.0), 200 mM NaCl, 10% glycerol, and 1 mM TCEP. The purified SMAD2–MAN1 and SMAD1–MAN1 complexes were concentrated to 7 mg/ml and 4 mg/ml, respectively, for crystallization.

For the binding assays, His-Trx-MAN1(762–890)-SDED and its mutants, His-Trx-MAN1(762–911)-SDED and its mutants, His-Trx-SMAD2C-2E and its mutants, His-GST-SMAD2C-2E, His-GST-SMAD1C-2E and its mutant, His-Trx-SKI(16–40)-SDED and His-Trx-CBP(1941-1973)-SDED were purified with the Ni-NTA Superflow resin (QIAGEN) using the same method as the SMAD2–MAN1 complex purification. The eluted proteins were further purified using a MonoQ HR 10/10 (GE Healthcare) column that was pre-equilibrated with buffer containing 10 mM Tris–HCl (pH 9.0), 10% glycerol and 1 mM TCEP, and the proteins were eluted using a linear gradient of 0-1 M NaCl. Trx-MAN1(762–890)-SDED, Trx-MAN1(762–911)-SDED, Trx-SMAD2C-2E, SMAD2C-2E and SMAD1C-2E were purified using a similar method. In short, the protein solutions after Ni-NTA purification were treated with HRV3C protease at 4°C overnight to cleave the N-terminal His-tag, and the treated solutions were purified using a MonoQ HR 10/10 column.

### Crystallization and structure determination

The concentrated SMAD2–MAN1 and SMAD1–MAN1 complexes were crystallized by the sitting-drop vapor-diffusion method. Crystals of the SMAD2–MAN1 complex were obtained in a reservoir solution containing 0.1 M acetate (pH 6.1), 7.5% PEG4000 and 10% 2-propanol at 4°C. Crystals of the SMAD1–MAN1 complex were obtained in a reservoir solution containing 0.1 M MES (pH 6.9) and 10% PEG20000 at 20°C. The X-ray diffraction dataset of the SMAD2–MAN1 complex crystal was collected at beamline AR-NE3A of the Photon Factory (Tsukuba, Japan) under cryogenic conditions (95 K). For cryoprotection, the SMAD2–MAN1 crystal was soaked in reservoir solution supplemented with 40% ethylene glycol for a few seconds. The X-ray diffraction dataset of the SMAD1–MAN1 complex crystal was collected in-house using a FR-E SuperBright and an R-AXIS VII (Rigaku) under cryogenic conditions (93 K). For cryoprotection, the SMAD1–MAN1 crystal was soaked in reservoir solution supplemented with 30% ethylene glycol for a few seconds. The X-ray diffraction data were indexed, integrated and scaled with XDS (30). The crystals of the SMAD2–MAN1 and SMAD1–MAN1 complexes diffracted X-rays to resolutions of 2.79 and 2.85 Å, respectively. The SMAD2–MAN1 complex crystal belonged to the space group *P*6_5_ with the unit cell parameters *a* = *b* = 176.81 Å and *c* = 73.85 Å. The SMAD1–MAN1 complex crystal belonged to the space group *P*4_1_32 with the unit cell parameters *a* = *b* = *c* = 187.05 Å. The initial models of the SMAD2–MAN1 and the SMAD1–MAN1 complexes were determined by the molecular replacement method using the program MOLREP (31) in the CCP4 suite (32) using the coordinates of the SMAD2 MH2 domain (PDB code: 5XOD) (8) and the SMAD1 MH2 domain (PDB code: 1KHU) (33), respectively. The initial models were refined and rebuilt using the program Phenix.refine (34) and Coot (35). The geometries of the final structures were evaluated using the program Molprobity (36). The data collection and refinement statistics of the SMAD2–MAN1 and SMAD1–MAN1 complexes are summarized in Table 1.

**Table 1.** Summary of data collection and refinement statistics of the SMAD2–MAN1 and SMAD1–MAN1 complexes

	SMAD2–MAN1	SMAD1–MAN1
Data collection		
Space group	*P*6_5_	*P*4_1_32
Cell dimensions		
*a, b, c* (Å)	176.81, 176.81, 73.85	187.05, 187.05, 187.05
Resolution (Å)	45.55–2.79 (2.94–2.79)*	46.76–2.85 (3.00–2.85)
*R*_sym_ (%)	13.6 (97.8)	9.1 (73.3)
Mean (*I*/σ*I*)	8.2 (1.1)	15.5 (2.2)
Completeness (%)	96.9 (92.8)	99.6 (98.6)
Redundancy	5.4 (3.6)	6.0 (5.9)
CC (1/2)	0.994 (0.531)	0.998 (0.735)
Refinement		
Resolution (Å)	42.47–2.79	42.91–2.85
*R/R*_free_ (%)	24.19/26.92	21.49/25.49
No. atoms		
SMAD1/2	4451	3198
MAN1	1932	2015
*B*-factors (Å^2^)		
SMAD1/2	75.06	61.44
MAN1	86.31	84.84
RMSD		
Bond lengths (Å)	0.002	0.003
Bond angles (^o^)	0.574	0.618
Ramachandran plot		
Favored region (%)	97.0	96.6
Allowed region (%)	100	100

*The numbers in parentheses represent data for the highest-resolution shells.

### Pull-down assay

For the pull-down assay of MAN1(762–890) and its mutants with SMAD2, His-Trx-MAN1(762–890)-SDED or its mutants (2.5 μM), Trx-SMAD2C-2E (5 μM) and the Ni-NTA Superflow resin were mixed in 50 mM Tris–HCl (pH 9.0), 200 mM NaCl, 10 mM imidazole, 10% glycerol and 1 mM TCEP. For the pull-down assay of MAN1(762–911) and its mutants with SMAD2, His-Trx-MAN1(762–911)-SDED or its mutants (2.5 μM), Trx-SMAD2C-2E (5 μM) and the Ni-NTA Superflow resin were mixed in the same buffer. For the pull-down assay of SMAD2 and its mutants with MAN1, His-Trx-SMAD2C-2E or its mutants (5 μM), Trx-MAN1(762–890)-SDED (5 μM) and the Ni-NTA Superflow resin were mixed in the same buffer. For the pull-down assay of SMAD1 and its mutant with MAN1, His-GST-SMAD1C-2E or its mutant (2.5 μM), Trx-MAN1(762–890)-SDED (5 μM) and the Ni-NTA Superflow resin were mixed in the same buffer. For the pull-down assay of MAN1 with SMAD2 and SMAD1, His-GST-SMAD2C-2E or His-GST-SMAD1C-2E (2.5 μM), Trx-MAN1(762–890)-SDED (5, 10 and 20 μM) and the Ni-NTA Superflow resin were mixed in the same buffer. For the cooperative binding assay of MAN1 and SKI, His-Trx-SKI(16–40)-SDED (10 μM), Trx-SMAD2C-2E or Trx-SMAD2C-2E P377A (10 μM), Trx-MAN1(762–890)-SDED (20 μM) and Ni-NTA Superflow resin were mixed in the same buffer. For the cooperative binding assay of MAN1 and CBP, His-Trx-CBP(1941-1973)-SDED (7.5 μM), Trx-SMAD2C-2E or Trx-SMAD2C-2E P377A (7.5 μM), Trx-MAN1(762–890)-SDED (15 μM) and Ni-NTA Superflow resin were mixed in the same buffer. The proteins remaining on the resin were then washed with 50 mM Tris–HCl (pH 9.0), 200 mM NaCl, 20 mM imidazole, 10% glycerol and 1 mM TCEP, and the bound proteins were eluted with 50 mM Tris–HCl (pH 9.0), 200 mM NaCl, 200 mM imidazole, 10% glycerol and 1 mM TCEP. The eluted solutions were separated by SDS-PAGE and were visualized by Coomassie staining. For the pull-down assay of the SMAD2 and MAN1 mutants, the densities of the protein bands were quantified with the ImageJ software (National Institutes of Health). The concentrations of the Trx-SMAD2C-2E and Trx-MAN1(762–890)-SDED bands were normalized to those of the His-Trx-MAN1(762–890)-SDED and His-Trx-SMAD2C-2E bands in the same lane of the SDS-PAGE gel, respectively.

### Co-immunoprecipitation assay

To construct full-length MAN1 expression vectors used in mammalian cells, full-length human MAN1 cDNA was amplified by PCR and was subcloned into EcoRI and XhoI sites of pcDNA3-HA vector. Vector pcDNA3-HA-MAN1 was digested with BamHI and XhoI, and the C-terminal 0.5-kb fragment of MAN1 was removed. The mutated versions of the C-terminal fragments were amplified by PCR using His-Trx-MAN1 expression vectors as the templates with the following primers (BamHI-hMAN1 2237-2305: 5′-ggtggatccagccttctgcatcctgtgacaaaatattagttataccttctaaagtatggcaaggtcaag-3′, XhoI-hMAN1 2736-2646:

5′-atgctcgagtcaggaacttccttgagaattggttaggccagtccgaagacgaagatgagacatggagttcatatgtttatttgatggcttcaatggagtg-3′) and were subcloned into the BamHI and XhoI sites of pcDNA3-HA-MAN1. For the full-length human SMAD1 and SMAD2 vectors, pcDNA3-Flag-human SMAD1 and pcDNA3-Flag-human SMAD2 vectors (37) were digested with HpaI and XhoI, and C-terminal 0.45-kb fragments were removed. The mutated versions of the C-terminal fragments were amplified by PCR using His-GST-SMAD1C-2E, His-GST-SMAD2C-2E, or His-Trx-SMAD2C-2E expression vectors as the templates and the following primers (pGEX5′: 5′-gggctggcaagccacgtttggtg-3′, XhoI-Stop-Smad1E Cterm: 5′-cgagctcgagttactctacctctgaaatagg-3′, T7: 5′-taatacgactcactataggg-3′, XhoI-Stop-Smad2E Cterm: 5′-cgagctcgagtcactccatctctgagc-3′) and were subcloned into the HpaI and XhoI sites of pcDNA3-FLAG-SMAD1 or SMAD2. The resulting SMAD1 and SMAD2 vectors expressed the C-terminal phosphorylated mimic versions (SEXE) of the SMAD proteins (SMAD1E and SMAD2E).

HEK293 cells were cultured in DMEM supplemented with 10% FBS and penicillin/streptomycin at 37°C in a humidified atmosphere of 5% CO_2_. These cells were transfected with the aforementioned plasmids, using PEI-MAX (Polysciences) and lysed 48 h after transfection and processed as described previously (8). For immunoprecipitation, a rat monoclonal anti-HA antibody (3F10, Roche) was used. For the detection of proteins, a mouse Flag antibody (M2, Sigma) and a horseradish peroxidase-conjugated anti-HA antibody (3F10, Roche) were used.

### Luciferase assay

Luciferase assay was done as described previously (8).

### Thermal shift assay

For the thermal shift assay of SMAD1 and SMAD2, SMAD1C-2E (10 μM) or SMAD2C-2E (10 μM), Trx-MAN1(762–911)-SDED (10 μM) and ×2.5 SYPRO Orange (Thermo Fisher Scientific) were mixed in 10 mM Tris–HCl (pH 9.0), 200 mM NaCl and 1 mM TCEP. Thermal shift assays were performed using a CFX Connect Real-Time PCR Detection System (Bio-Rad Laboratories). Fluorescence was measured from 20 to 95°C in 0.5°C steps (excitation, 450–490 nm; detection, 560–580 nm). Data are analyzed using the Bio-Rad CFX Manager 3.0 software.

### Isothermal titration calorimetry (ITC)

ITC experiments were performed using a MicroCal iTC_200_ isothermal titration calorimeter (GE Healthcare) at 20°C. SMAD1C-2E, SMAD2C-2E and Trx-MAN1(762–890)-SDED were dialyzed against 100 mM Na_2_HPO_4_ (pH 8.0), 150 mM NaCl and 1 mM TCEP. SMAD1C-2E (20 μM) and SMAD2C-2E (25 μM) in the sample cell (204 μl) were titrated by 18 injections of 2 μl of Trx-MAN1(762–890)-SDED (255 μM). The first injection of 0.4 μl was ignored in the final data analysis. Data were analyzed using the program Origin. Binding parameters were calculated with a ‘one set of sites’ model.

### Computational analysis

The structures of the SMAD2–MAN1 and SMAD1–MAN1 complexes were analyzed using the following set of computer programs: PISA for the analysis of the protein interface, surface and assemblies (38); Clustal Omega for the amino acid sequence alignment (39); ESpript for the preparation of alignment figures (40); DISOPRED2 for the prediction of disordered region (41); Dali for the search for similar structures from the database (42); APBS for the calculation of macromolecular electrostatics (43); and Pymol (https://www.pymol.org/) for the depiction of the structures.

## RESULTS

### Structure determination of R-SMAD–MAN1 complexes

**Figure 1.**
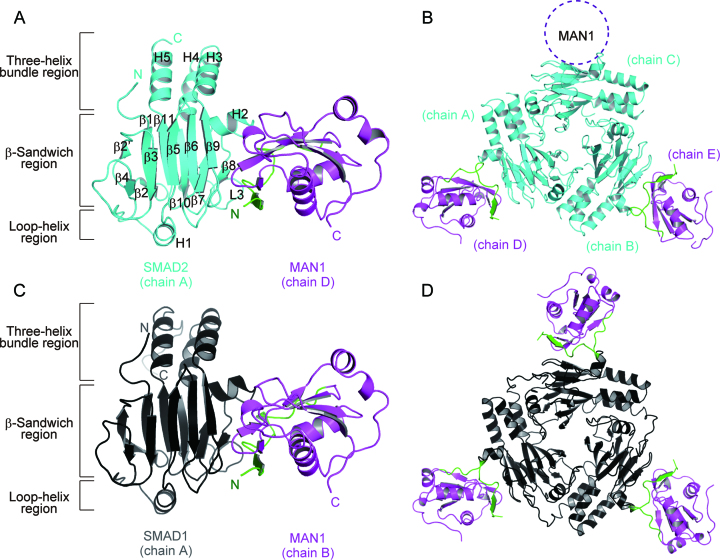
Overall structure of the SMAD2–MAN1 and SMAD1–MAN1 complexes. (**A**) The monomeric structure of SMAD2 in complex with MAN1. SMAD2, the MAN1 ULM and the MAN1 UHM domain are coloured cyan, green and purple, respectively. Secondary structure assignments of SMAD2 are labelled on the model. (**B**) The SMAD2–MAN1 complex structure in the asymmetric unit. The predicted additional MAN1 binding region is indicated by a dotted circle. (**C**) The monomeric structure of SMAD1 in complex with MAN1. SMAD1 is coloured black. (**D**) The trimeric structure of SMAD1 (chain A) in complex with MAN1 (chain B) generated by a crystallographic three-fold axis.

R-SMAD binding by MAN1 requires the UHM domain (residues 782–890), its N-terminal ULM (residues 758–781) and the C-terminal disordered region (residues 891–911), which is less conserved among the homologues (Supplementary Figures S1B and S2C) (26). The ULM, especially Trp765 and Qln766, is essential for R-SMAD binding. By contrast, the C-terminal disordered region is not critical for R-SMAD binding, although it is involved in the binding (26). To obtain crystals of human SMAD2–MAN1 and SMAD1–MAN1 complexes that were suitable for X-ray crystallography experiments, we used a MAN1 construct that did not contain the C-terminal disordered region (residues 762–890) for co-crystallization experiments. This MAN1 region interacted with both SMAD1 and SMAD2 (Supplementary Figure S3A). Co-crystals of the SMAD2–MAN1 complex were obtained using an MH2 domain of SMAD2, in which the C-terminal phosphorylation region was truncated (residues 262–458, SMAD2C-dC) (Supplementary Figure S2A). Co-crystals of the SMAD1–MAN1 complex were obtained using the phosphorylated-state mimics of the MH2 domain of SMAD1 (residues 259–465 (S463E and S465E), SMAD1C-2E) (Supplementary Figure S2B). The SMAD2–MAN1 and SMAD1–MAN1 complex structures were determined at resolutions of 2.79 and 2.85 Å, respectively. The final model of the SMAD2–MAN1 complex structure contains three SMAD2 protomers (chains A, B and C) that form a trimeric structure, although we used SMAD2 lacking the C-terminal phosphorylation region that is needed for the trimerization of SMAD2, and two MAN1 molecules (chains D and E) that bind to SMAD2 protomers (Figure 1A, B and Supplementary Figure S4A–C). The structure of SMAD2 in the SMAD2–MAN1 complex is similar to those of pre-existing SMAD2 structures; the SMAD2 structure in the SMAD2–MAN1 complex is composed of the three-helix bundle region, the β-sandwich region and the loop-helix region. The maximal root mean square deviation (RMSD) between the structures of SMAD2 in the SMAD2–MAN1 complex and those in the SMAD2-SMAD4 complex (PDB code: 1U7V) (44) is 0.55 Å for 185 superposed Cα atoms (Supplementary Figure S5A). The electron density map indicated that each SMAD2 binds one MAN1 at its β-sandwich region. However, due to the poor electron density, we could not build a structure model for one of the three MAN1 molecules (Figure 1B). This leads to the relatively high *R*_free_ value of the SMAD2–MAN1 structure (Table 1). The SMAD1–MAN1 complex contains two SMAD1 protomers (chains A and B) and two MAN1 molecules (chains C and D) in its asymmetric unit and forms two SMAD1–MAN1 complexes (Figure 1C, D and Supplementary Figure S4D–F). Each SMAD1 forms a trimeric structure with symmetrically related SMAD1 structures generated by a crystallographic three-fold axis. The SMAD1 structure in the SMAD1–MAN1 complex is composed of the three-helix bundle region, the β-sandwich region and the loop-helix region similar to other R-SMAD structures. The maximal RMSD between the structures of SMAD1 in complex with MAN1 and those in the cofactor-free form (PDB code: 1KHU) (33) is 0.59 Å for 194 superposed Cα atoms (Supplementary Figure S5B). These data indicate that the main chain structures of SMAD2 and SMAD1 are not modified by the binding of MAN1. The structures of the β-sandwich regions of SMAD2 and SMAD1 that are used for MAN1 binding are also similar to that of the monomeric state of SMAD3 (PDB ID: 1MJS) (11). The maximal RMSD between the structures of the β-sandwich regions of SMAD2 and SMAD1 in complex with MAN1 and that of the monomeric SMAD3 is 0.56 Å for 139 superposed Cα atoms (Supplementary Figure S5C, D).

### MAN1 structure

**Figure 2.**
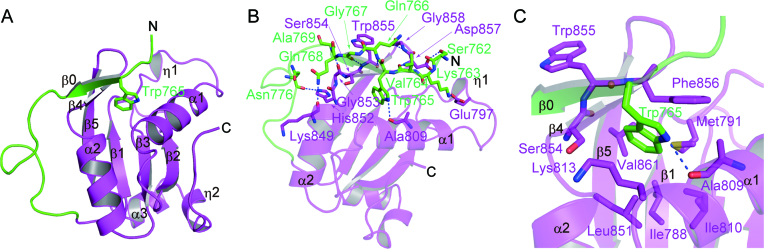
Intramolecular UHM–ULM interaction in MAN1. (**A**) Structure of the UHM–ULM interaction of MAN1. The ULM and UHM domain are coloured green and purple, respectively. Secondary structure assignments are labelled on the model. The conserved tryptophan residue in the ULM (Trp765) is shown as a stick model. (**B**) Intramolecular UHM–ULM interaction in MAN1. Residues that form the intramolecular UHM–ULM interaction are shown as stick models. Hydrogen bonds are shown as blue dotted lines. (**C**) Trp765 recognition pocket of the MAN1 UHM domain. Trp765 and its interacting residues are shown as stick models.

The UHM domain structure of MAN1 consists of five β strands, three α helices and two 3_10_ (η) helices and adopts the typical RRM-family βαββαβ fold (Figure 2A). The five β strands form a curved antiparallel β sheet (β4–β5–β1–β3–β2). The concave face of the β sheet binds helices α1 and α2, and the convex face of the sheet binds helices η2 and α3. The UHM domain is a non-canonical RRM that is used for intermolecular interactions with other proteins that contain ULM. The ULM is characterized by an invariant tryptophan and its N-terminal positively charged residues (26,45). A previous study has predicted that the MAN1 UHM domain forms an intramolecular UHM–ULM interaction with the MAN1 ULM that contains conserved Trp765 and positively charged Lys763 (26). The MAN1 ULM (residues 762–781) interacts with the MAN1 UHM domain at the protein surface of helices α1, α2, η1 and strand β4 (Figure 2A, B). A typical UHM–ULM interaction shows that the positively charged residues of ULM interact with a negatively charged helix α1 of the UHM domain (45). However, the side chain of Lys763 of the MAN1 ULM forms a salt bridge with Glu797 of the η1–α1 loop, although the α1 of the MAN1 UHM domain also possesses a negatively charged surface (Supplementary Figure S6). The side chain of the invariant Trp765 is accommodated in a narrow hydrophobic pocket that consists of Ile788, Met791, Ala809, Ile810, Lys813, Leu851, Ser854, Phe856 and Val861, and forms a hydrogen bond with the main chain carbonyl of Ala809 (Figure 2C). The intramolecular UHM–ULM interaction of MAN1 is further stabilized by eight hydrogen bonds (Figure 2B). Using these hydrogen bonds, the residues 765 to 768 of ULM form a β strand (β0) that is antiparallel to the β4 strand of the UHM domain. The interfacial area between the ULM and UHM domains of MAN1 is approximately 792 Å^2^. The structure of Trp855 on the β4 strand seems to be stabilized by this intramolecular UHM–ULM interaction (Figure 2C). The function of the side chain of Trp855 will be discussed later. A database search using the Dali server (42) showed that the MAN1 UHM domain structure determined in this study shows the highest structural similarity to the UHM domain of the RNA-binding protein 39 (RBM39, PDB code: 5CXT, *Z*-score = 17.5, RMSD = 1.9 Å, sequence identity = 39%) (46).

The WH domain and the UHM domain of MAN1 are used to bind to the *BMAL1* promoter to modulate the circadian rhythmicity (47). A previous study has indicated that the UHM domain of MAN1 enhances the DNA-binding properties of the WH domain (25). The electrostatic potential of the MAN1 UHM domain surface shows that MAN1 possesses a positively charged surface among Lys787, Lys864, Arg870 and Arg874 (Supplementary Figure S6), which corresponds to the RNA-binding surface of RRM (27). However, the RNA-stacking residues of RRM are not conserved in the MAN1 UHM domain (Supplementary Figure S1B). This positively charged surface is also not used for SMAD2 and SMAD1 binding (Figure 1). This positively charged surface might interact with the negatively charged DNA backbone to enhance MAN1-DNA interaction.

### SMAD2–MAN1 interaction

**Figure 3.**
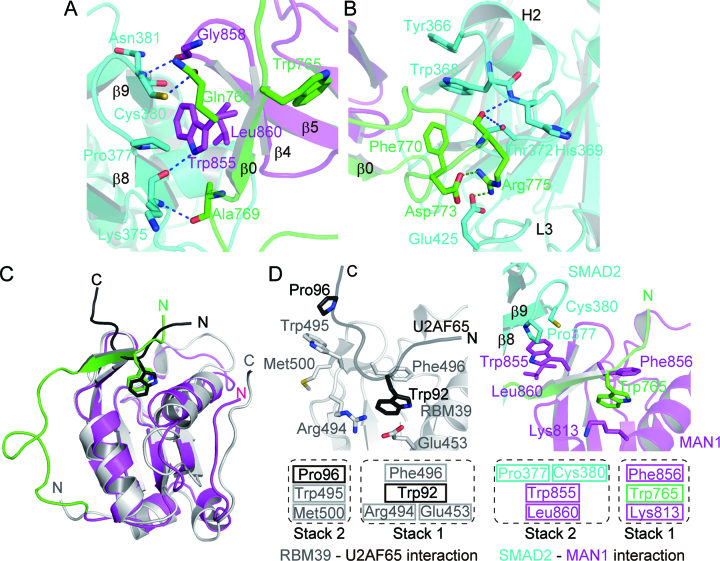
SMAD2–MAN1 interaction. (**A**) MAN1 recognition by SMAD2 at strands β8 and β9. SMAD2, the MAN1 ULM and the MAN1 UHM domain are coloured cyan, green and purple, respectively. Hydrogen bonds are shown as blue dotted lines. (**B**) MAN1 recognition by SMAD2 around the H2 helix and the L3 loop. Salt bridges are shown as green dotted lines. (**C**) Superposition of the structures of MAN1 and the RBM39 (grey)-U2AF65 (black) complex. Conserved tryptophan residues in ULM are shown as stick models. (**D**) Structure comparison between the SMAD2–MAN1 complex and the RBM39-U2AF65 complex.

The SMAD2–MAN1 complex structure shows that the SMAD2 MH2 domain binds MAN1 using the hydrophobic surface of the H2 helix, the strands β8 and β9, and the L3 loop (Figure 1A). The interfacial area between SMAD2 and MAN1 (the A-D interface of the SMAD2–MAN1 complex) is approximately 593 Å^2^. The Trp855, which is stabilized by the intramolecular UHM–ULM interaction of MAN1 (Figure 2C), is the key residue for the SMAD2–MAN1 interaction (Figure 3A). The side chain of Trp855 forms a stacking interaction with the hydrophobic surface of SMAD2 that is composed of Pro377 and Cys380, and with MAN1 Leu860. This hydrophobic interaction is further stabilized by four hydrogen bonds. The side chain of MAN1 Trp855 forms a hydrogen bond with the main chain carbonyl oxygen of SMAD2 Lys375, and Gln766, Ala769 and Gly858 of MAN1 form hydrogen bonds with Cys380, Lys375 and Asn381 of SMAD2, respectively. A previous study has shown that two hydrophobic residues of SMAD2 (Tyr366 and Trp368) are involved in the MAN1 recognition (29). In this region, the hydrophobic side chain of MAN1 Phe770 stacks with Trp368 and Tyr366 of SMAD2 (Figure 3B). In addition, the side chain of MAN1 Arg775 forms salt bridges with SMAD2 Glu425 and MAN1 Asp773, and the main chain of Arg775 forms hydrogen bonds with SMAD2 His369 and Thr372.

Typical UHM domains are used for intermolecular interactions with other proteins that possess ULM. However, the intramolecular UHM–ULM interaction observed in the SMAD2–MAN1 structure is used for the intermolecular interaction with R-SMAD proteins. The structural comparison of the MAN1 UHM–ULM complex with the RBM39 (UHM)-U2AF65 (ULM) complex (46) shows that each UHM domain accommodates a tryptophan residue of ULM at the same site (Figure 3C). In the RBM39-U2AF65 complex, the UHM–ULM interaction is stabilized by two stacking interactions (Figure 3D). At the tryptophan binding pocket of the RBM39 UHM, Trp92 of U2AF65 stacks with Phe496 and with a salt bridge between Glu453 and Arg494 of RBM39 (stack 1). Pro96 of U2AF65 stacks with Trp495 and Met500 of RBM39 (stack 2). These stacking interactions are a common feature of the UHM–ULM interactions (45,46). In the SMAD2–MAN1 complex, Trp765 of the MAN1 ULM stacks with Lys813 and Phe856 of the MAN1 UHM domain, similar to the structure of the RBM39-U2AF65 complex (stack 1); Lys813 and Phe856 of the MAN1 UHM domain correspond to the Glu453-Arg494 salt bridge and Phe496 of RBM39, respectively. By contrast, in the SMAD2–MAN1 complex, the second stacking interaction is used for the SMAD2 recognition; Leu860 and Trp855 of MAN1 stack with the hydrophobic surface of Pro377 and Cys380 of SMAD2 to stabilize the SMAD2–MAN1 complex (stack 2) (Figure 3D).

### SMAD1–MAN1 structure

**Figure 4.**
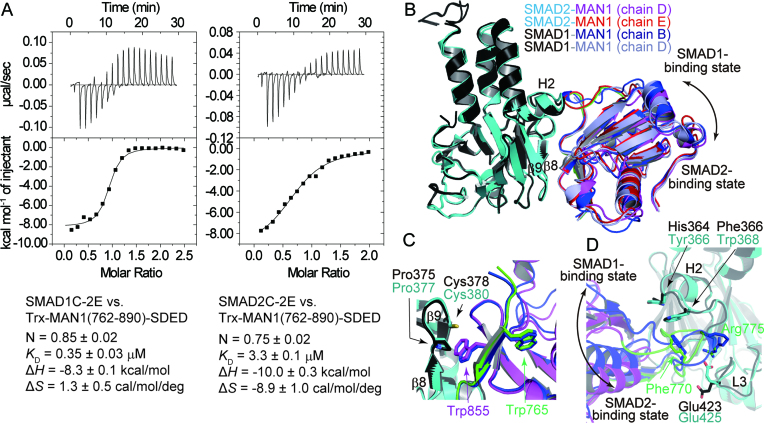
SMAD1–MAN1 complex structure. (**A**) Binding curves obtained by ITC experiments. SMAD1C-2E and SMAD2C-2E were titrated with Trx-MAN1(762–890)-SDED. Data are mean ± SEM from triplicate experiments. The images are representative of triplicate experiments. (**B**) Superposition of the structures of SMAD2 and SMAD1 in complex with MAN1. SMAD2 and SMAD1 are coloured cyan and black, respectively. The ULM and the UHM domains of MAN1 (chain D) in complex with SMAD2 are coloured green and purple, respectively. MAN1 (chain E) in complex with SMAD2 is coloured red. MAN1 in complex with SMAD1 (chains B and D) are coloured blue and light blue, respectively. (**C**) Structure comparison of the SMAD2–MAN1 (chains A and D) and SMAD1–MAN1 (chains A and B) complexes around the strands β8 and β9 of SMAD2. The structures are superposed using the coordinates of SMAD2 and SMAD1. SMAD2 and SMAD1 residues are labelled by cyan and black texts, respectively. MAN1 residues are labelled by green (ULM) and purple (UHM) texts. (**D**) Structure comparison of SMAD2–MAN1 (chains A and D) and SMAD1–MAN1 (chains A and B) complexes around the H2 helix and the L3 loop of SMAD2. The structures are superposed using the coordinates of SMAD2 and SMAD1.

In *Xenopus*, the C-terminal region of MAN1 binds SMAD1, SMAD5 and SMAD8 more strongly than SMAD2 (48). The pull-down assay also showed that human MAN1(762–890) bound to SMAD1C-2E more strongly than SMAD2C-2E (Supplementary Figure S3A). The thermal shift assay showed that the melting temperature of SMAD1C-2E (*T*_m_ = 48.5°C) was increased by the addition of Trx-MAN1(762–911)-SDED (*T*_m_ = 50.5°C), although that of SMAD2C-2E (*T*_m_ = 49.0°C) was not changed by the addition of Trx-MAN1(762–911)-SDED. This result also suggests that MAN1 binds to SMAD1C-2E more strongly than SMAD2C-2E (Supplementary Figure S3B–D). ITC experiments showed that Trx-MAN1(762–890)-SDED bound to SMAD1C-2E and SMAD2C-2E with dissociation constants of 0.35 and 3.3 μM, respectively (Figure 4A). These results indicate that the UHM–ULM region of human MAN1 prefer to bind to the MH2 domain of SMAD1 rather than that of SMAD2. To analyze the SMAD1 preference mechanism of MAN1, we also determined the SMAD1–MAN1 complex structure. The SMAD1–MAN1 complex structure shows that the MAN1 binding mechanism of SMAD1 is approximately the same as that of SMAD2; the SMAD1 MH2 domain binds MAN1 using the hydrophobic surface of the H2 helix, the strands β8 and β9, and the L3 loop (Figures 1C and 4B). When the R-SMAD–MAN1 complex structures are superposed using their SMAD structures, the positions of MAN1 differ slightly among the complexes (Figure 4B). These differences may indicate that the binding of MAN1 by the R-SMAD proteins is relatively flexible. The interfacial area between SMAD1 and MAN1 (the A–B interface of the SMAD1–MAN1 complex) is approximately 620 Å^2^. The SMAD1–MAN1 interaction using the intramolecular UHM–ULM interaction of MAN1 is approximately the same as that observed in the SMAD2–MAN1 complex (Figure 4C). By contrast, the hydrogen bonds and salt bridges around Arg775 of MAN1 in the SMAD2–MAN1 complex are not observed in the SMAD1–MAN1 complex (Figure 4D).

The MAN1 binding surface is highly conserved between SMAD2 and SMAD1 except for Tyr366 and Trp368 of SMAD2; Tyr366 and Trp368 of SMAD2 are substituted for His364 and Phe366 in SMAD1, respectively (Supplementary Figure S1A). Although Pro378 and Asn381 of SMAD2, which is also used for MAN1 binding, are not conserved between SMAD2 and SMAD1, these residues interact with MAN1 using their main chain atoms. By contrast, the MAN1 binding residues of R-SMAD proteins are not conserved in Co-SMAD (SMAD4) and inhibitory SMAD (I-SMAD: SMAD6 and SMAD7) (Supplementary Figure S1C), indicating that MAN1 is specific for R-SMAD proteins. When the structure of SMAD2–MAN1 complex and that of SMAD1–MAN1 complex are compared, the distances between the MAN1 Phe770 and the MH2 domains of SMAD2 and SMAD1 are different (Figure 4D); the minimum side chain distance between the MAN1 Phe770 and the SMAD2 Trp368 is 3.7 Å (between chains B and E in the SMAD2–MAN1 complex), although that between the MAN1 Phe770 and the SMAD1 Phe366 is 3.3 Å (between chains A and B in the SMAD1–MAN1 complex). This difference suggests that the hydrophobic core between SMAD1 and MAN1 is more compact than that between SMAD2 and MAN1. This difference may contribute to the SMAD1 preference of MAN1.

### Mutation assay

**Figure 5.**
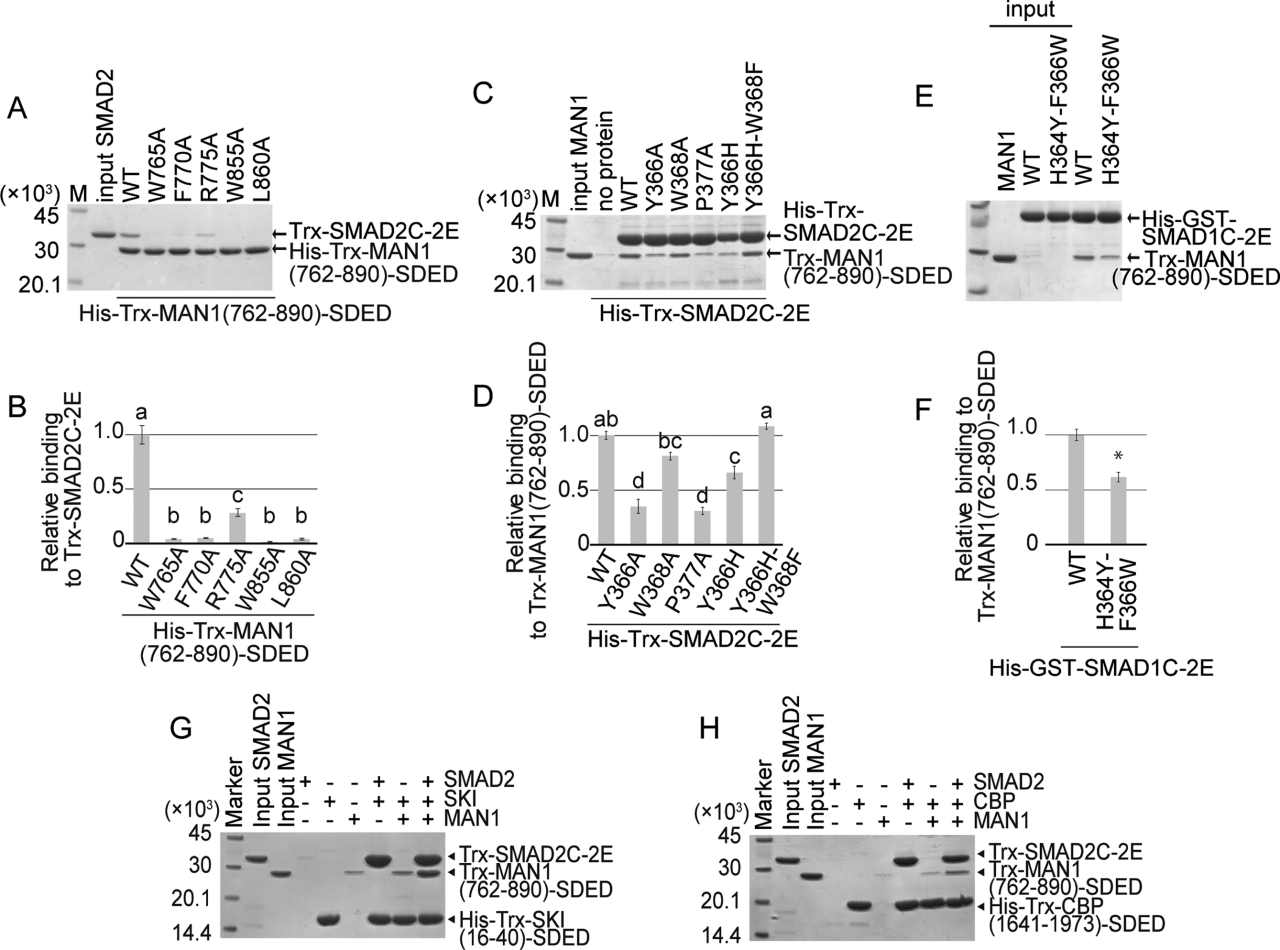
Binding assay. (**A**) The electrophoretic pattern of SMAD2 (Trx-SMAD2C-2E) and MAN1 (His-Trx-MAN1(762–890)-SDED and its mutants) after a His-tag pull-down assay. The sizes of the protein markers (lane M) are indicated on the left side of the panel. (**B**) The relative affinities of the MAN1 mutants. Data are mean ± SEM from triplicate experiments. Bars sharing the same letter are not significantly different (one-way analysis of variance (ANOVA) with Tukey's multiple comparison test, *P* < 0.05). (**C**) The electrophoretic pattern of SMAD2 (His-Trx-SMAD2C-2E and its mutants) and MAN1 (Trx-MAN1(762–890)-SDED) after a His-tag pull-down assay. (**D**) The relative affinities of SMAD2 mutants. Data are mean ± SEM from triplicate experiments. Bars sharing the same letter are not significantly different (one-way ANOVA with Tukey's multiple comparison test, *P* < 0.05). (**E**) The electrophoretic pattern of SMAD1 (His-GST-SMAD1C-2E and its mutant) and MAN1 (Trx-MAN1(762–890)-SDED after a His-tag pull-down assay. (**F**) The relative affinity of a SMAD1 mutant. Data are mean ± SEM from triplicate experiments. *P < 0.05 compared to wild type (WT) by two-tailed Student's *t*-test. (**G, H**) Interaction of SMAD2 (Trx-SMAD2C-2E) with MAN1 (Trx-MAN1(762–890)-SDED) and SKI (His-Trx-SKI(16–40)-SDED) (G) or CBP (His-Trx-CBP(1941-1973)-SDED) (H). After the His-tag pull-down assay, the proteins were separated by SDS-PAGE. The gel images are representative of triplicate experiments.

To analyze the importance of the interacting residues of SMAD2 and MAN1, we created alanine mutants at positions Trp765, Phe770, Arg775, Trp855 and Leu860 in MAN1 to evaluate the importance of their side chains and analyzed their SMAD2 binding abilities by pull-down assay (Figure 5AB and Supplementary Figure S7). Trp765 of MAN1 is a key residue for the intramolecular UHM–ULM interaction of MAN1. The SMAD2 binding ability of MAN1 was highly reduced by the W765A mutation. The hydrophobic surface of MAN1 consisting of Trp855 and Leu860, which is stabilized by the intramolecular UHM–ULM interaction, is used for the SMAD2 binding. The W855A and L860A mutants also showed reduced SMAD2 binding abilities. In addition, the mutation of MAN1 Phe770, which stacks with Tyr366 and Trp368 of SMAD2, to alanine also reduced the SMAD2 binding ability of MAN1. By contrast, the R775A mutant showed only moderately reduced activity, although Arg775 of MAN1 forms hydrogen bonds and a salt bridge with SMAD2. These results suggest that the SMAD2–MAN1 interaction is mostly hydrophobic, and Arg775 of MAN1 is not so important for R-SMAD binding. In fact, Arg775 does not form any contact with SMAD1 in the SMAD1–MAN1 complex (Figure 4D).

We also created alanine mutants at positions Tyr366, Trp368 and Pro377 in SMAD2 to evaluate the importance of their side chains and analyzed their MAN1 binding abilities by pull-down assay (Figure 5C, D). Tyr366 and Trp368 of SMAD2 stack with Phe770 of MAN1 to stabilize the SMAD2–MAN1 complex. A previous study has also shown that Tyr366 and Trp368 of SMAD2 are required for MAN1 binding (29). The MAN1 binding ability of SMAD2 was reduced by the Y366A mutation. However, the W368A mutant of SMAD2 did not show significantly reduced MAN1 binding ability. The mutation of Pro377, which stacks with Trp855 of MAN1, also reduced the MAN1 binding ability of SMAD2. The UHM–ULM region of MAN1 binds to the MH2 domain of SMAD1 more strongly than that of SMAD2 (Figure 4A and Supplementary Figure S3). The SMAD2–MAN1 structure showed that the MAN1 binding residues of SMAD2 are highly conserved in SMAD1, excepting Tyr366 and Trp368 (Supplementary Figure S1A); Tyr366 and Trp368 of SMAD2 are substituted for histidine (His364) and phenylalanine (Phe366) in SMAD1, respectively. To analyze the differences of these residues between SMAD1 and SMAD2, we also created the Y366H mutant and the Y366H–W368F double mutant of SMAD2 and analyzed their MAN1 binding abilities by pull-down assay (Figure 5C, D). We could not produce W368F mutant of SMAD2 due to its poor solubility. The Y366H mutant of SMAD2 showed a reduced MAN1 binding ability. However, the MAN1 binding ability of SMAD2 was recovered by the Y366H-W368F double mutation. In addition, the H364Y-F366W double mutant of SMAD1 showed a reduced MAN1 binding ability (Figure 5E, F). These results suggest that the pair of histidine and tyrosine residues at this position (His364 and Phe366 of SMAD1) are important for the SMAD1 preference of MAN1, although the other residues would also be critical in the SMAD1 preference of MAN1.

To consolidate the results of *in vitro* pull-down assay, we performed co-immunoprecipitation assay with full-length version of SMAD and MAN1 mutants transiently expressed in HEK293 cells. As shown in Supplementary Figure S8AB, Phe770, Trp855 and Leu860 of the full-length MAN1 protein are crucial amino acids to bind both full-length SMAD1 and SMAD2 proteins. We also demonstrated that Tyr366 and Pro377 of SMAD2 are the key residues for the interaction between full-length of MAN1 and SMAD2 proteins (Supplementary Figure S8C, D). Furthermore, luciferase assays revealed that the MAN1 mutants that do not bind to wild type full-length SMAD2 failed to suppress TGF-β/activin-dependent activation of a SMAD-specific luciferase reporter (12xCAGA-luc) (Supplementary Figure S8EF). Similarly, full-length MAN1 mutants that do not bind to wild type full-length SMAD1 failed to suppress BMP-dependent activation of a BMP–SMAD-specific luciferase reporter (BRE-luc) (Supplementary Figure S8G). Therefore, Phe770, Trp855 and Leu860 of MAN1, which were elucidated by crystal structural analysis, are proved to be functionally indispensable amino acid residues to inhibit SMAD-dependent signaling.

### Comparison with other R-SMAD–cofactor complexes

**Figure 6.**
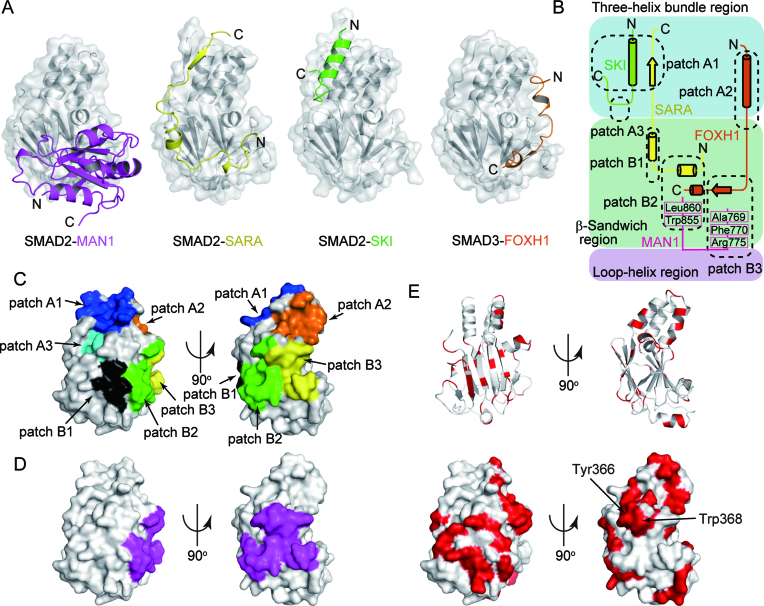
Cofactor bindings by R-SMAD proteins. (**A**) The structures of the SMAD2- and SMAD3-cofactor complexes. The SMAD2 and SMAD3 structures are shown as grey surface models. The bound MAN1 (UHM domain and ULM), SARA (SMAD binding domain), SKI (R-SMAD binding domain) and FOXH1 (SMAD interaction motif) are shown as purple, yellow, green and orange cartoon models, respectively. (**B**) A schematic diagram of the hydrophobic patches of the MH2 domain of SMAD2 and SMAD3. Cyan, green and pink regions represent the three-helix bundle region, the β-sandwich region and the loop-helix region, respectively. (**C**) Cofactor binding patches on the MH2 domain of SMAD2. The patches A1, A2, A3, B1, B2 and B3 are coloured blue, orange, cyan, black, green and yellow, respectively. (**D**) The MAN1 binding surface of SMAD2 is coloured purple. (**E**) Sequence conservation of the MH2 domains of R-SMAD proteins. The SMAD2 residues and surface that are not conserved among R-SMAD proteins are coloured red.

Thus far, the structures of SMAD2-SARA (10), SMAD3-SARA (11), SMAD2-SKI (8) and SMAD3-FOXH1 (8) complexes have been determined as R-SMAD–cofactor complexes. Our previous study has shown that the MH2 domain of SMAD2 and SMAD3 possesses multiple hydrophobic patches on its surface (patches A1 to A3 and B1 to B3), and SMAD cofactors tether to the patches to bind to SMAD2 and SMAD3 in a cooperative or competitive manner (Figure 6A–C and Table 2) (8). The structures of the SMAD2–MAN1 and SMAD1–MAN1 complexes show that MAN1 tethers to the patches B2 (hydrophobic surface of the H2 helix and the β8 and β9 strands) and B3 (cleft between the H2 helix and the L3 loop) of SMAD2 and the corresponding regions of SMAD1 using Ala769, Phe770, Arg775, Trp855 and Leu860 (Figure 6B–D). The cofactor binding patch B2 of SMAD2 and SMAD3 binds the rigid coil structure of SARA (10,11) and the C-terminal hydrophobic helix of FOXH1 (8) (Figure 6A, B). The cofactor binding patch B3 of SMAD2 and SMAD3 binds the conserved Pro-Pro-Asn-Lys-Ser sequence of the FOXH1 SMAD interaction motif (SIM) (Figure 6A, B) (8). MAN1 and other SMAD cofactors that interact with patches B2 and B3 would compete for binding to SMAD2 and SMAD3. Actually, MAN1 and FOXH1 compete for SMAD2 binding in the cell (29). Meanwhile, because the MAN1 binding regions are independent from patches A1 (hydrophobic surface of the helices H3 and H5) and A3 (small pocket between the three-helix bundle region and the β-sandwich region), which are used for transcription coactivator and corepressor binding (8), MAN1 is predicted to be able to bind to R-SMAD proteins cooperatively with transcription coactivators and corepressors that bind to patches A1 and A3. The pull-down assay showed that the transcriptional coactivator CBP (residues 1941 to 1973) and the transcriptional corepressor SKI (residues 16–40) could bind to SMAD2 cooperatively with MAN1 (Figure 5G, H). These cooperative bindings are disrupted by a point mutation at position Pro377 in SMAD2, which is located at patch B2, to alanine, although CBP and SKI could bind to the SMAD2 P377A mutant (Supplementary Figure S9A, B). These results indicate that the MAN1 binding site of SMAD2 is independent of the CBP and SKI binding site (Supplementary Figure S9C).

**Table 2.** Hydrophobic patches of SMAD2 and SMAD3

	Patch ID	Location	Binding Cofactors*	Sequence conservation
Three-helix bundle region	A1	Hydrophobic surface of the helices H3 and H5	SKI, SARA	Conserved in all R-SMAD proteins
	A2	Hydrophobic surface of the helices H2, H3 and H4	FOXH1	SMAD2 and SMAD3 specific
	A3	Small pocket between the three-helix bundle region and the β-sandwich region	SKI	Conserved in all R-SMAD proteins
				
β-sandwich region and loop-helix region	B1	Hydrophobic surface of the strands β5 and β6	SARA	SMAD2 and SMAD3 specific
	B2	Hydrophobic surface of the H2 helix and the β8 and β9 strands	MAN1, SARA, FOXH1	Conserved in all R-SMAD proteins excepting Tyr366 and Trp368 of SMAD2
	B3	Cleft between the H2 helix and the L3 loop	MAN1, FOXH1	Conserved in all R-SMAD proteins

*SMAD2–SKI complex (PDB ID: 5XOD) (8); SMAD2–SARA complex (PDB ID: 1DEV) (10); SMAD3–SARA complex (PDB ID: 1MK2) (11); SMAD3–FOXH1 complex (PDB ID: 5XOC) (8).

Although SMAD2 and SMAD3 bind SARA and FOXH1 using the same site as MAN1, the structural bases for SMAD2 and SMAD3 bindings of these cofactors are not conserved (Figure 6A). In the SMAD2–SARA and the SMAD3–FOXH1 complex structures, patch B2 of SMAD2 and SMAD3 recognizes the hydrophobic helices of SARA and FOXH1 (8,10,11). By contrast, MAN1 binds to patch B2 of SMAD2 using the hydrophobic surface on the β sheet that is stabilized by the intramolecular UHM–ULM interaction (Figure 3A). At patch B3 of SMAD3, the Pro-Pro-Asn-Lys-Ser sequence of FOXH1 is accommodated in the cleft between the helix H2 and the L3 loop, and the sequence forms the β strand that is parallel to the β8 strand of SMAD2 (8). By contrast, in the structures of the SMAD2–MAN1 complex, the β0–β1 loop of MAN1, which is antiparallel to the β8 strand of SMAD2, is accommodated in the H2-L3 cleft, and Phe770 of MAN1 forms a hydrophobic contact with Trp368 of SMAD2 (Figure 3B). These structures show that the cofactors that bind to the same hydrophobic patch of the R-SMAD proteins do not necessarily possess conserved structural motifs. Previous studies have also shown that patch A1 of SMAD2 interacts with both the β strand of SARA (10,11) and the amphiphilic a helix of SKI (8).

## DISCUSSION

R-SMAD proteins are central transcription factors of TGF-β superfamily signaling in cells and form many transcription factor complexes with SMAD cofactors. Each SMAD cofactor binds to either SMAD2 and SMAD3 or SMAD1, SMAD5 and SMAD8, or both to regulate TGF-β superfamily signal-dependent gene expression. MAN1 is one of the SMAD cofactors and forms complexes with both SMAD2 and SMAD3, and SMAD1, SMAD5 and SMAD8 to terminate TGF-β/Nodal/Activin and BMP signaling pathways in the cell (22,23,29). In this study, we determined the crystal structures of the SMAD2–MAN1 and SMAD1–MAN1 complexes and analyzed the structural basis for the R-SMAD recognition mechanism by MAN1. The complex structures show that MAN1 uses the intramolecular UHM–ULM interaction to bind to the hydrophobic surface of the H2 helix, the strands β8 and β9, and the L3 loop of the MH2 domains of SMAD2 and SMAD1 (the patches B2 and B3 of SMAD2 and the corresponding region of SMAD1). Most UHM–ULM interactions are used for protein-protein interactions between pre-mRNA splicing factors (27). The intramolecular UHM–ULM interaction is not observed in other protein structures.

Previous studies have demonstrated that MAN1 weakly binds to protein phosphatase 1A (PPM1A), which dephosphorylates R-SMAD proteins (49,50), to inactivate TGF-β superfamily signaling at the nuclear envelope (29). In addition, PPM1A directly interacts with R-SMAD proteins (49,50). The structural similarity between SMAD2 in complex with MAN1 and that in complex with SMAD4 suggests that MAN1 binds to a SMAD2-SMAD2-SMAD4 heterotrimeric complex similarly to how it binds to SMAD2 in the SMAD2–MAN1 complex (Supplementary Figure S5A). Because the R-SMAD proteins form a heterotrimeric complex with SMAD4 using their phosphorylated SXS motif at their C-terminus, the local position of each phosphorylated SXS motif is different; one phosphorylated SXS motif interacts with the other R-SMAD protein, while the other phosphorylated SXS motif interacts with SMAD4 (Supplementary Figure S10). Because PPM1A interacts with both R-SMAD proteins and MAN1, PPM1A may interact with R-SMAD proteins not in the interfacial area between SMAD4 and R-SMAD but in the interfacial area between two R-SMAD proteins to dephosphorylate the C-terminal SXS motif (Supplementary Figure S10). However, clarification of the precise mechanisms by which PPM1A interacts with R-SMAD and MAN1 requires further structural studies on the PPM1A–R-SMAD and PPM1A-MAN1 complexes. MAN1 has also been demonstrated to inactivate TGF-β superfamily signaling at the nuclear envelope by competing with transcription factors that bind to R-SMAD proteins. MAN1 binds to patches B2 and B3 of SMAD2 and the corresponding region of SMAD1 (Figure 6B). These cofactor binding patches are used for FOXH1 binding and are also predicted to be used for Mixer binding (8), indicating that MAN1 inactivates signals mediated by the SMAD2- and SMAD3-FOXH1 complexes and by the SMAD2- and SMAD3-Mixer complexes. By contrast, MAN1 and SMAD2 enhance *BMAL1* transcription, which modulates the circadian rhythm (47). Because the MAN1 binding surface of SMAD2 is independent from the transcription coactivator and corepressor binding surface of SMAD2 (patches A1 and A3), SMAD2 could bind a transcriptional coactivator or corepressor simultaneously with MAN1 (Figure 5G, H). R-SMAD coactivators or corepressors may modulate *BMAL1* regulation by MAN1.

Many SMAD cofactors specifically bind to either the phosphorylated state or non-phosphorylated state of R-SMAD proteins. For example, the transcription factor FOXH1 preferentially binds to the phosphorylated (heterotrimeric) SMAD2 (19). In addition, the transcriptional coactivator CBP and the corepressor SKI also preferentially bind to the phosphorylated (heterotrimeric) state of R-SMAD proteins (11,51–53). SARA and ENDOFIN bind to the non-phosphorylated (monomeric) states of R-SMAD proteins (10–12). By contrast, MAN1 is a SMAD cofactor that binds to R-SMAD proteins in a signal-independent manner; MAN1 binds to both phosphorylated and non-phosphorylated R-SMAD proteins (22). A comparison of the SMAD structures shows that the structure of the three-helix bundle region is modified by the homo- and heterotrimer formation, indicating that the structures of patches A1, A2 and A3 of SMAD2 and SMAD3, and the corresponding region of SMAD1, SMAD5 and SMAD8 are modified by the trimer formation (Supplementary Figure S5C, D). The structures of SMAD2-SARA (10), SMAD3-SARA (11), SMAD2-SKI (8) and SMAD3-FOXH1 (8) complexes show that SARA, SKI and FOXH1 bind to the three-helix bundle region of SMAD2 and SMAD3. By contrast, the structures of the SMAD2–MAN1 and SMAD1–MAN1 complexes show that MAN1 only uses the β-sandwich region whose structure is not modified by the trimer formation (Supplementary Figure S5C, D). SMAD cofactors that bind to the three-helix bundle region are predicted to bind to either the monomeric or trimeric state of R-SMAD proteins, whereas SMAD cofactors that only bind to the β-sandwich region are predicted to bind to both the monomeric and trimeric states of R-SMAD proteins.

The MH2 domains of R-SMAD proteins show approximately 74% amino acid sequence identity to one another (Supplementary Figure S1A). Each SMAD cofactor that binds to the MH2 domains of R-SMAD proteins recognizes this difference to select its binding partner. The amino acid sequence alignment of R-SMAD proteins shows that the patches A1, A3, B2 and B3 (except for Tyr366 and Trp368 of SMAD2) are conserved among the R-SMAD proteins. By contrast, the residues that compose the patches A2 and B1 are not conserved among R-SMAD proteins (Figure 6C–E, and Table 2). The sequence conservation of R-SMAD proteins suggests that cofactors that bind to every R-SMAD proteins use the patches A1, A3, B2 and B3 of SMAD2 and SMAD3, and the corresponding region of SMAD1, SMAD5 and SMAD8. Among the SMAD cofactors, transcription coactivators (CBP and p300) (14), corepressors (SKI and TGIF) (15–17) and MAN1 (22) bind to every R-SMAD protein. The structures of the SMAD2-SKI and SMAD2–MAN1 complexes show that SKI and MAN1 uses patches A1 and A3, and patches B2 and B3, respectively, to bind to SMAD2 and SMAD3 (8). By contrast, other SMAD cofactors, such as SARA and FOXH1, interact with SMAD2 and SMAD3 but not with SMAD1, SMAD5 and SMAD8 (10,19). The SMAD2-SARA complex structure shows that SARA uses the patch B1 and two hydrophobic residues (Tyr366 and Trp368) of patches B2 and B3 to bind to SMAD2 and SMAD3 (10,11). The SMAD3-FOXH1 complex structure shows that FOXH1 uses patch A2 to bind to SMAD2 and SMAD3 (Figure 6A, B) (8). SMAD cofactors that bind to patches A2 and B1 of SMAD2 and SMAD3 could be specific for SMAD2 and SMAD3. Understanding the cofactor selection mechanisms of R-SMAD proteins would reveal the crosstalk between TGF-β/Nodal/Activin signaling that is mediated by SMAD2 and SMAD3 and the BMP signaling that is mediated by SMAD1, SMAD5 and SMAD8.

## DATA AVAILABILITY

Atomic coordinates and structure factors for the reported crystal structures have been deposited with the Protein Data bank under accession number 5ZOJ (SMAD2–MAN1 complex) and 5ZOK (SMAD1–MAN1 complex).

## Supplementary Material

Supplementary Data
