# Supplementary material for: Structural basis for receptor-regulated SMAD recognition by MAN1
Source: Nucleic Acids Res. 2018 Oct 13;46(22):12139–53. doi: 10.1093/nar/gky925 (PMC6294489; doi:10.1093/nar/gky925)
Supplement: Supplementary Data [file gky925_supplemental_files.pdf]

Supplementary Materials

A

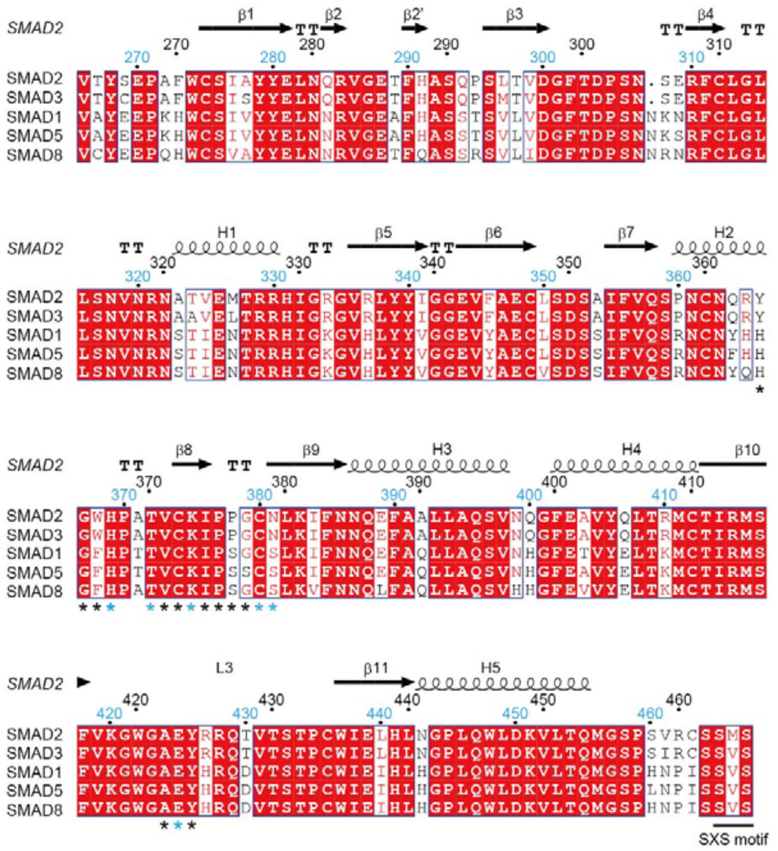

B

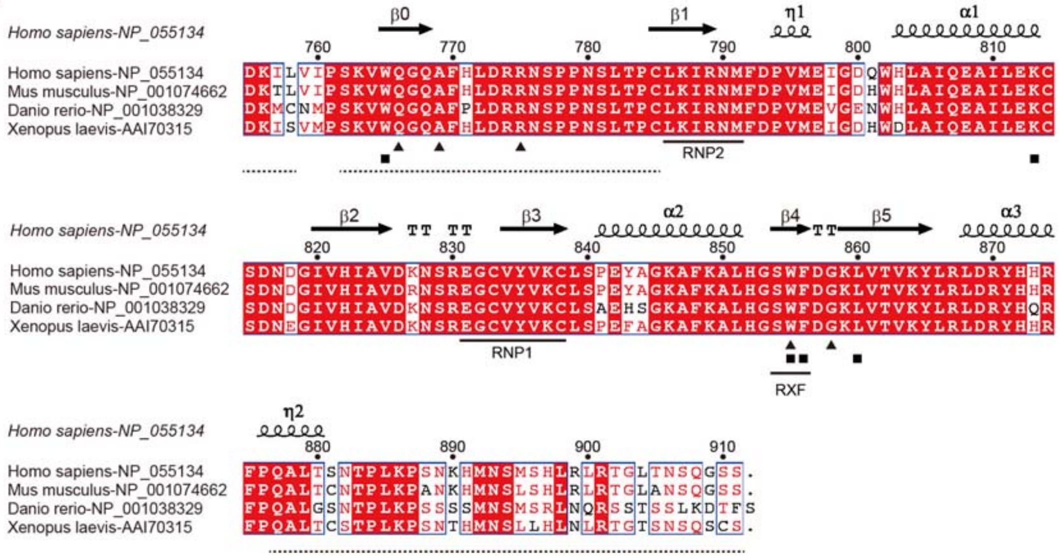

C

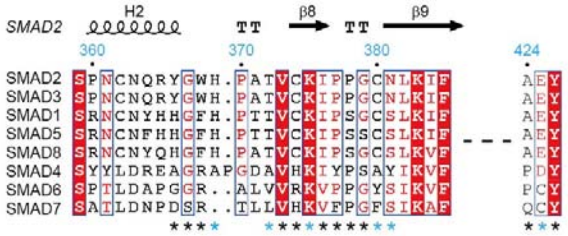

Supplementary Figure 1. Amino acid sequence alignment. (A) Amino acid sequence alignment of the MH2 domains of human R-SMAD proteins. The SXS motif conserved among R-SMAD proteins is indicated by a black line. The secondary structure of SMAD2 in the SMAD2-MAN1 complex (chain A) is indicated by helices (α helix), arrows (β strand) and TT (β turn). SMAD2 residues that are involved in SMAD2-MAN1 interactions are marked with asterisks. Residues that form hydrogen bonds with MAN1 are indicated by cyan asterisks. The residue numbers of SMAD2 and SMAD1 are shown as cyan and black texts, respectively. (B) Amino acid sequence alignment of the UHM-ULM regions of MAN1 orthologs. The secondary structure of MAN1 in the SMAD2-MAN1 complex structure (chain B) is indicated by helices (α- and η (3_{10})-helices), arrows (β-strand) and TT (β-turn). Residues that form hydrogen bonds with SMAD2 are marked with black triangles. Residues that form stacking interactions with SMAD2 are marked with closed squares. Residues that are predicted to be disordered are indicated by dotted lines. The conserved motifs of RRM (RNP2/RNP1/RXF) are indicated by black lines. The consensus sequences of RNP2, RNP1 and RXF motifs are [ILV]-[FY]-[ILV]-X-N-L, [RK]-G-[FY]-[GA]-[FY]-[ILV]-X-[FY] and R-X-F, respectively. X represents any amino acid. The RNA stacking residues of RRM are underlined. (C) Amino acid sequence alignment of human SMAD proteins around the MAN1 binding region. SMAD2 residues that are involved in SMAD2-MAN1 interactions are marked with asterisks. Residues that form hydrogen bonds with MAN1 are indicated by cyan asterisks.

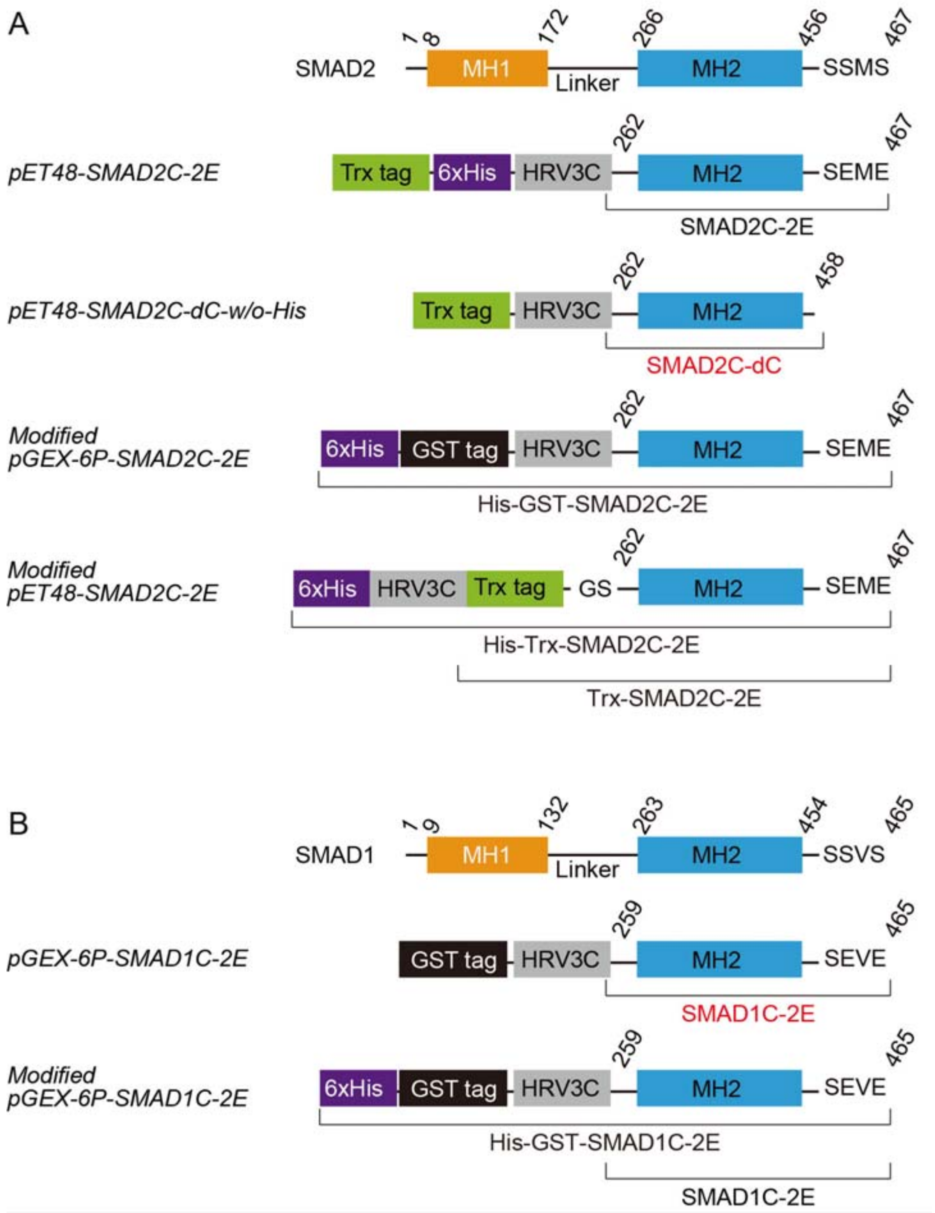
A
SMAD2
1
8
MH1
172
Linker
266
MH2
456
SSMS
467
pET48-SMAD2C-2E
Trx tag
6xHis
HRV3C
262
MH2
SEME
467
SMAD2C-2E
pET48-SMAD2C-dC-w/o-His
Trx tag
HRV3C
262
MH2
458
SMAD2C-dC
Modified pGEX-6P-SMAD2C-2E
6xHis
GST tag
HRV3C
262
MH2
SEME
467
His-GST-SMAD2C-2E
Modified pET48-SMAD2C-2E
6xHis
HRV3C
Trx tag
GS
262
MH2
SEME
467
His-Trx-SMAD2C-2E
Trx-SMAD2C-2E
B
SMAD1
1
9
MH1
132
Linker
263
MH2
454
SSVS
465
pGEX-6P-SMAD1C-2E
GST tag
HRV3C
259
MH2
SEVE
465
SMAD1C-2E
Modified pGEX-6P-SMAD1C-2E
6xHis
GST tag
HRV3C
259
MH2
SEVE
465
His-GST-SMAD1C-2E
SMAD1C-2E

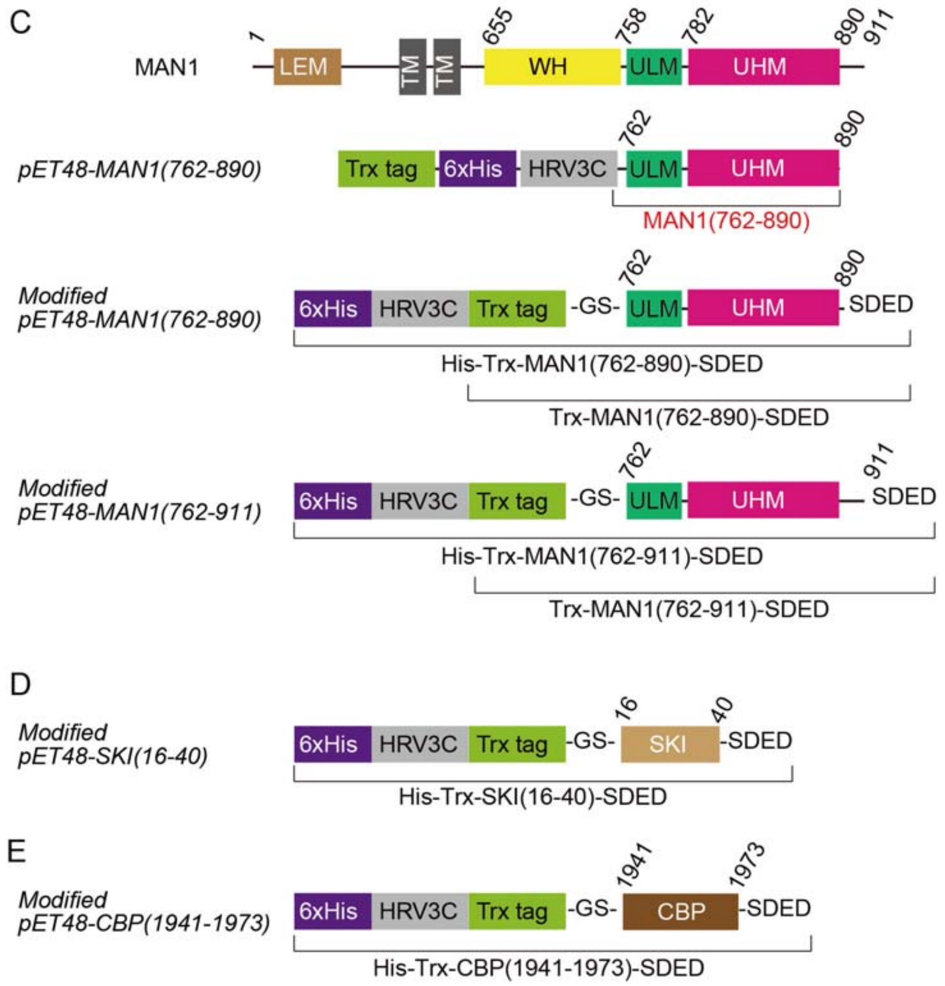

Supplementary Figure 2. Protein constructs. (A, B) SMAD2 (A) and SMAD1 (B) are multi-domain proteins that possess an N-terminal MAD homology 1 (MH1) domain and a C-terminal MH2 domain. The thioredoxin (Trx) tag, the 6×histidine (His) tag, the glutathione S-transferase (GST) tag and the HRV3C protease site are indicated as boxes. Plasmid names are shown by italic. Constructs used for the co-crystallization experiments are shown in red text. Constructs used for the biochemical assays are shown in black text. (C) MAN1 is a multi-domain protein that possesses an N-terminal LEM motif, transmembrane segments (TM), a winged-helix (WH) domain, a ULM and a C-terminal UHM domain. (D) The SKI constructs used in this report. (E) The CBP constructs used in this report.

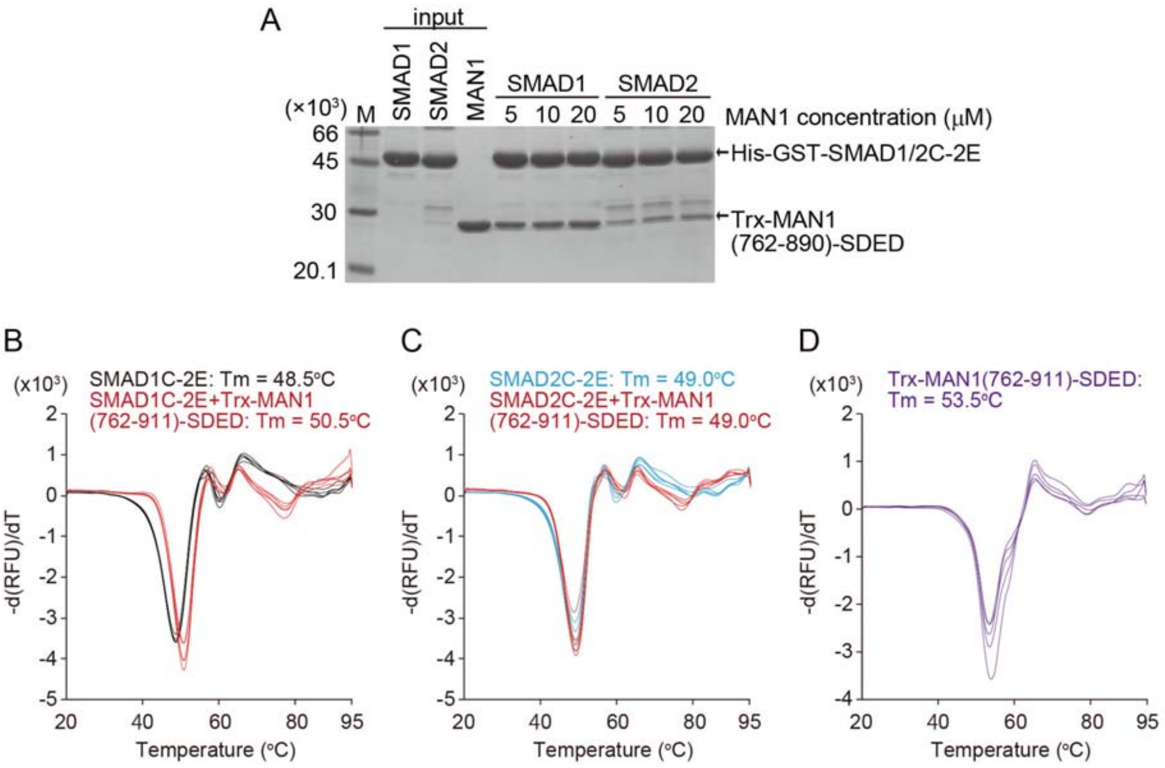

Supplementary Figure 3. SMAD1 and SMAD2 binding ability of MAN1. (A) Pull-down assay of MAN1 with SMAD2 and SMAD1. The electrophoretic pattern of SMAD1 (His-GST-SMAD1C-2E), SMAD2 (His-GST-SMAD2C-2E) and MAN1 (Trx-MAN1(762-890)-SDED) after a His-tag pull-down assay. The sizes of the protein markers (lane M) are indicated on the left side of the panel. The gel image is representative of triplicate experiments. (B) Thermal shift assay of SMAD1. The melting curves of SMAD1C-2E and its Trx-MAN1(762-911)-SDED complex are shown by black and red lines, respectively (*n* = 5). RFU, relative fluorescence unit. (C) Thermal shift assay of SMAD2. The melting curves of SMAD2C-2E and its Trx-MAN1(762-911)-SDED complex are shown by cyan and red lines, respectively (*n* = 5). (D) The Melting curves of Trx-MAN1(762-911)-SDED are shown by purple lines (*n* = 5).

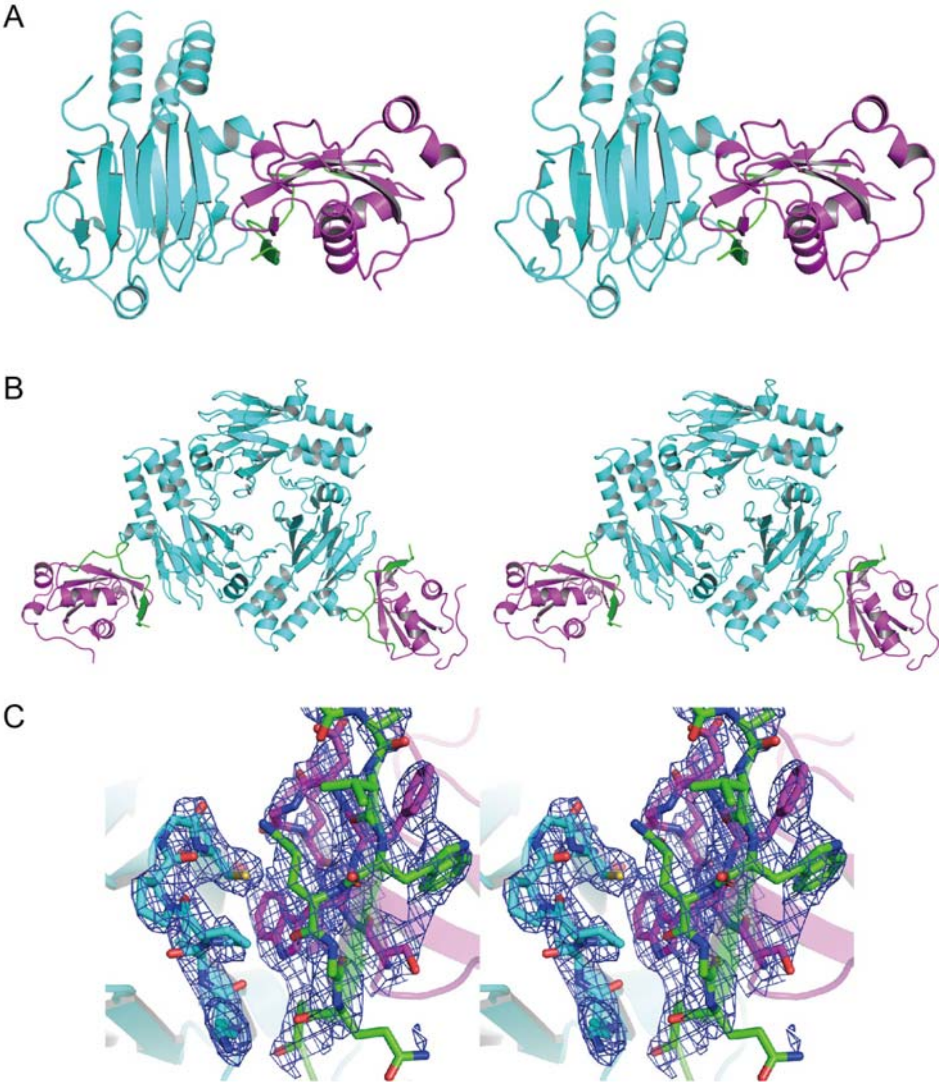
A
B
C

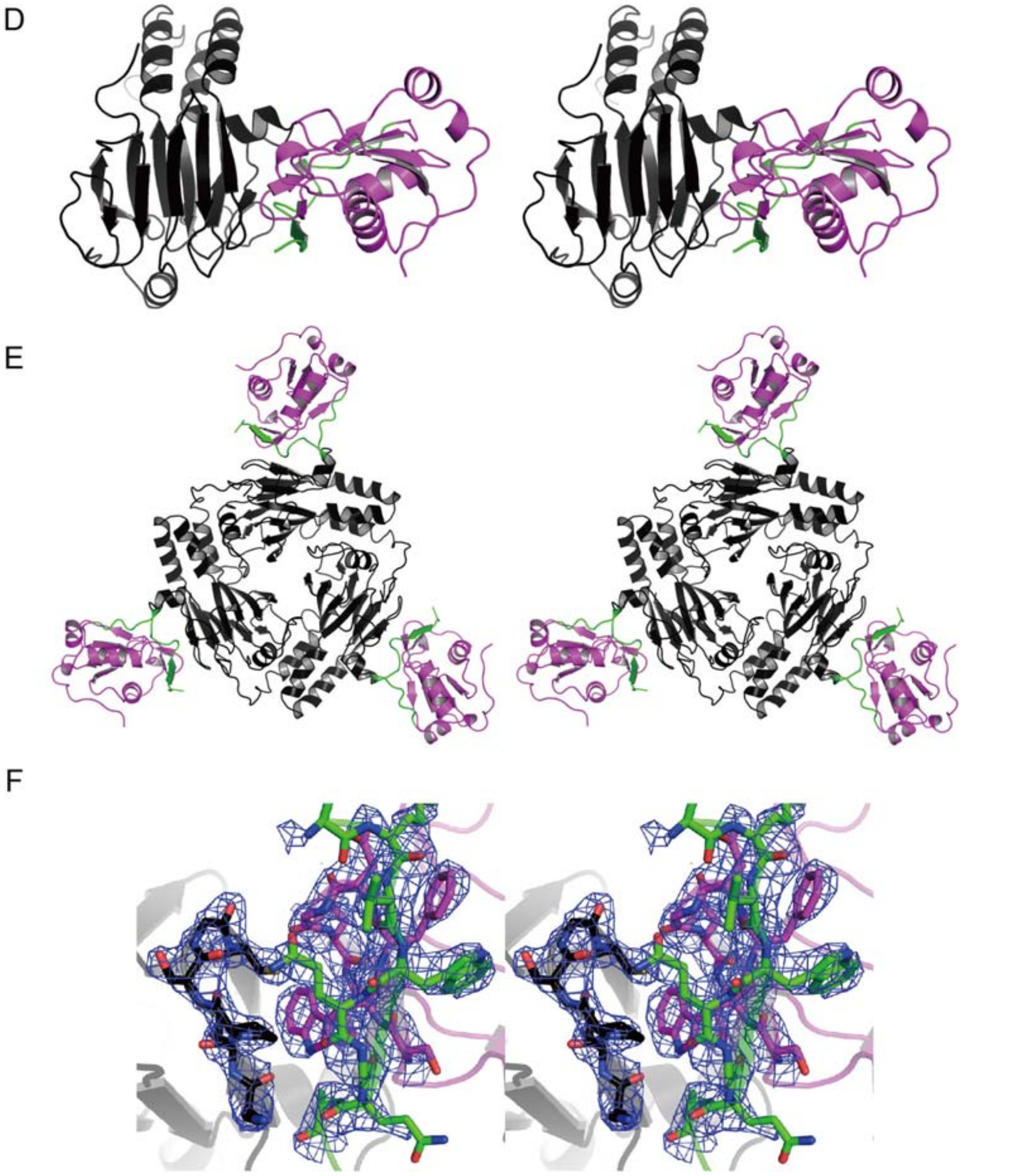

Supplementary Figure 4. Stereo diagram of the complex structures. (A) Stereo diagram of Figure 1A. (B) Stereo diagram of Figure 1B. (C) Stereo diagram of the SMAD2-MAN1 interface. The composite omit map is contoured at 1.5 σ (blue mesh). The composite omit map was generated using the CCP4 suite (32). (D) Stereo diagram of Figure 1C. (E) Stereo diagram of Figure 1D. (F) Stereo diagram of the SMAD1-MAN1 interface. The composite omit map is contoured at 1.5 σ (blue mesh).

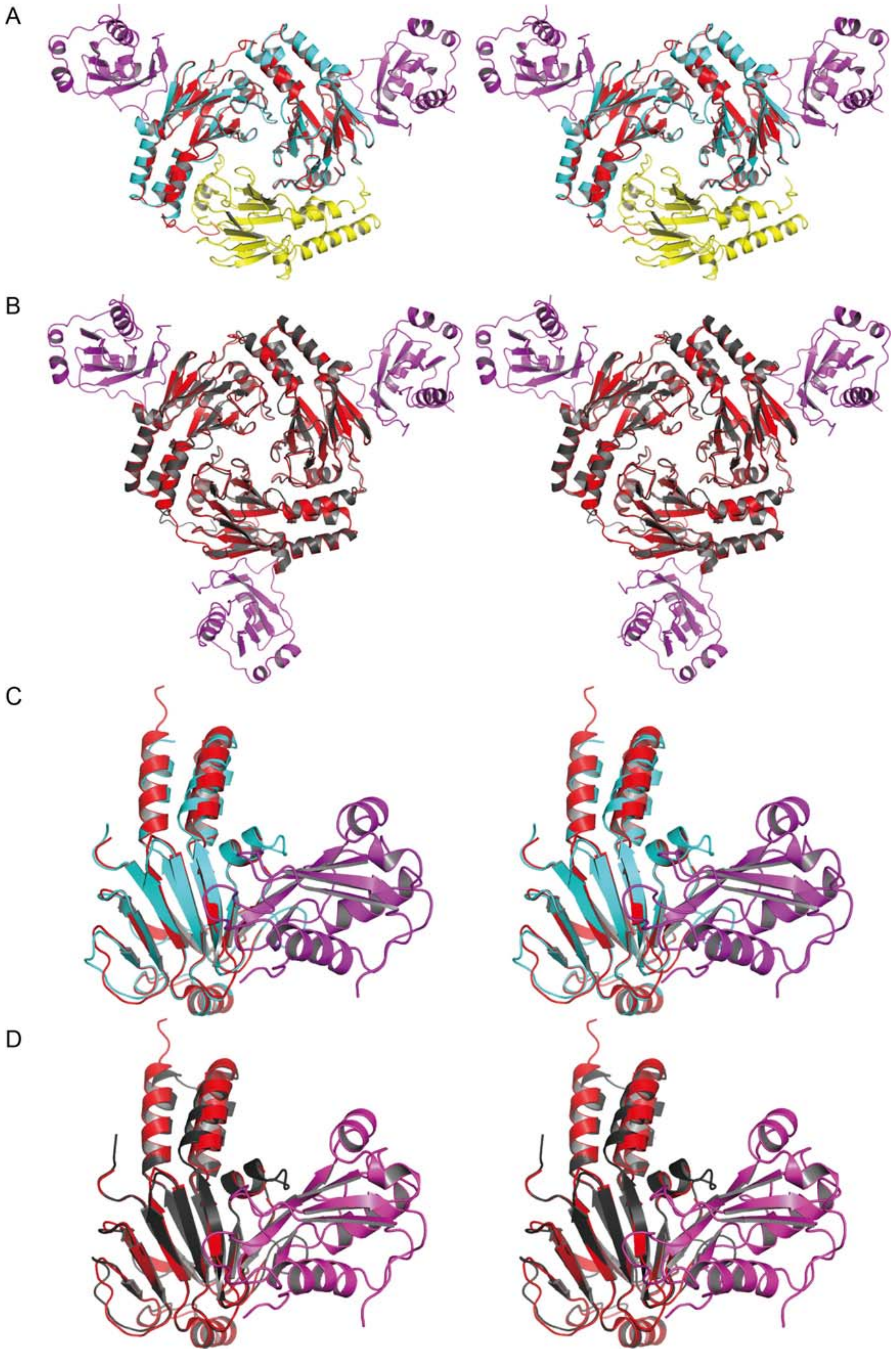
A
B
C
D

Supplementary Figure 5. Superposition of SMAD structures. (A) Stereo diagram of the superposition of the SMAD2 structures in the SMAD4 binding state (red) and the MAN1 binding state (cyan). SMAD4 and MAN1 are coloured yellow and purple, respectively. (B) Stereo diagram of the superposition of the SMAD1 structures in the cofactor-free state (red) and the MAN1-binding state (black). (C) Stereo diagram of the superposition of the structure of SMAD2 in complex with MAN1 and that of SMAD3 in the monomeric state (red). (D) Stereo diagram of the superposition of the structure of SMAD1 in complex with MAN1 and that of SMAD3 in the monomeric state (red).

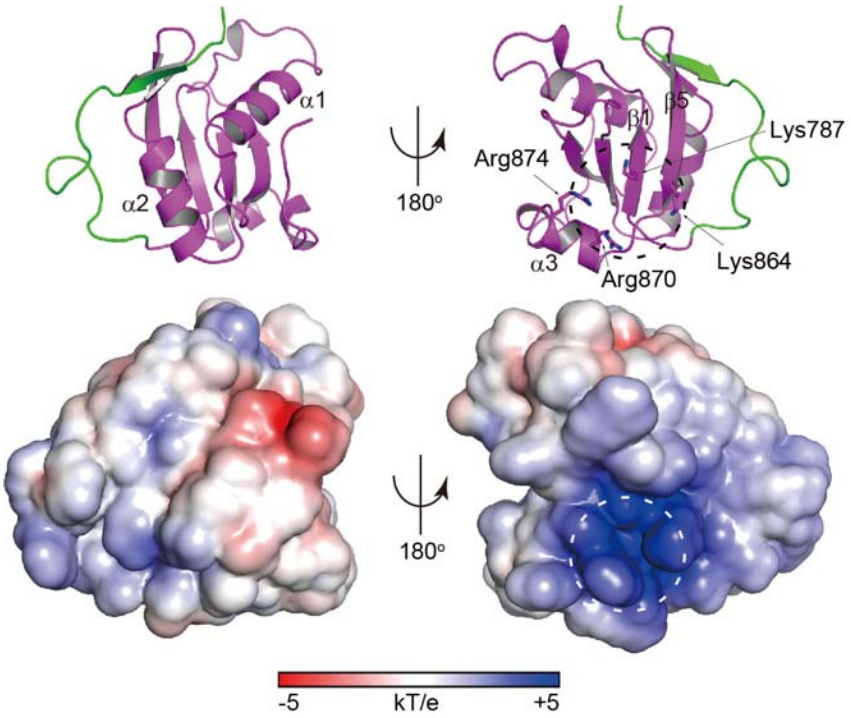

Supplementary Figure 6. Electrostatic potential of the MAN1 surface. The ±5 kT/e electrostatic potential of the MAN1 UHM-ULM region is plotted on the solvent-accessible surface. Positively charged surfaces are indicated by dotted circles. Positively charged residues on the β sheet are shown as stick models.

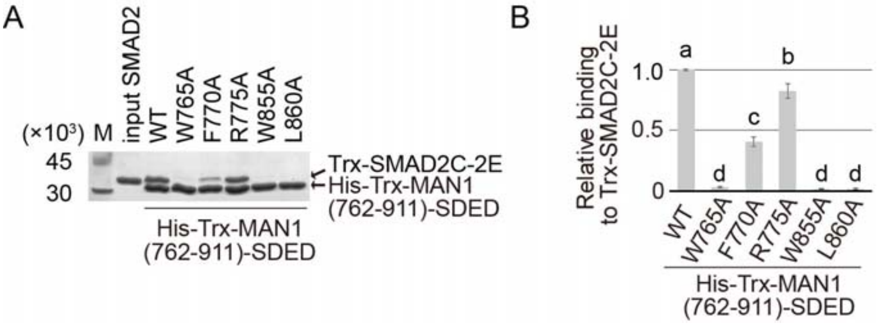

Supplementary Figure 7. Binding assay. (A) The electrophoretic pattern of SMAD2 (Trx-SMAD2C-2E) and MAN1 (His-Trx-MAN1(762-911)-SDED and its mutants) after a His-tag pull-down assay. The sizes of the protein markers (lane M) are indicated on the left side of the panel. The gel image is a representative of triplicate experiments. (B) The relative affinities of the His-Trx-MAN1(762-911)-SDED mutants. Data are mean ± SEM from triplicate experiments. Bars sharing the same letter are not significantly different (one-way analysis of variance (ANOVA) with Tukey’s multiple comparison test, $P < 0.05$).

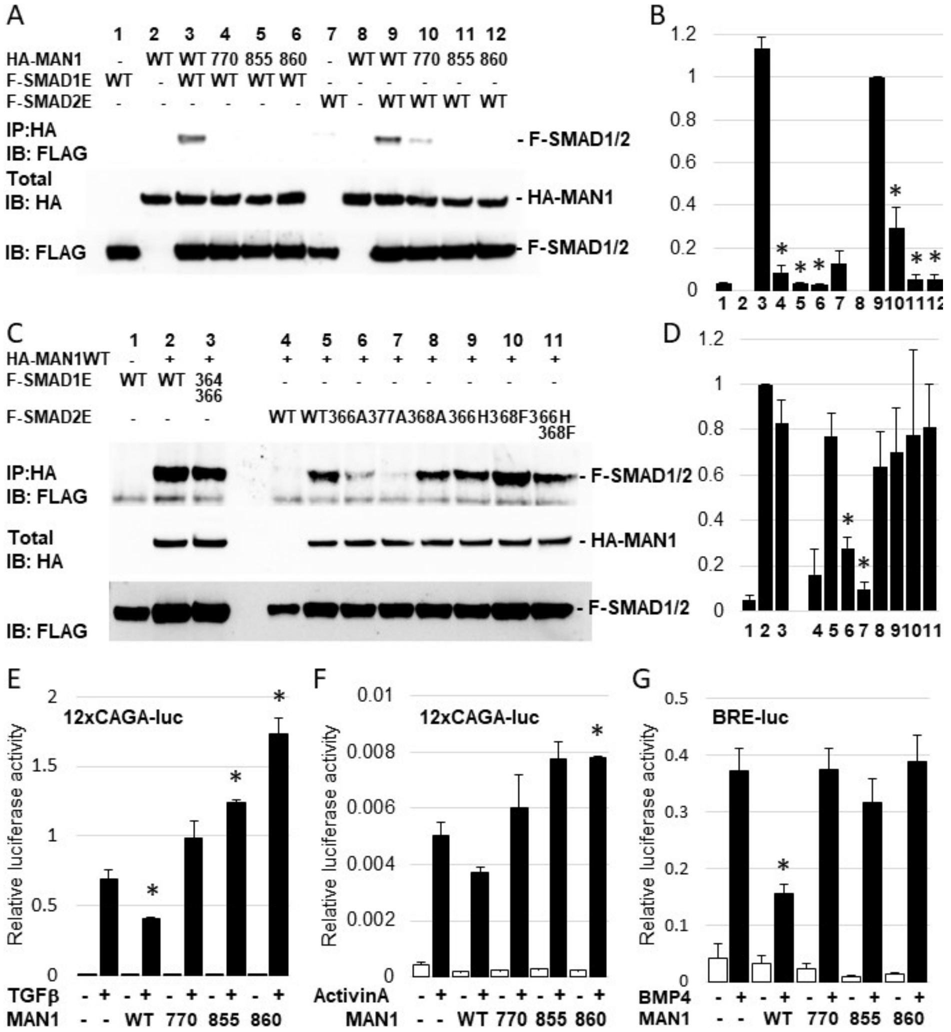

Supplementary Figure 8. Binding assay in cells. (A) Immunoblotting of full-length wildtype FLAG-SMAD1 and FLAG-SMAD2 with a combination of full-length HA-MAN1 mutants (F770A, W855A, and L860A) after the co-immunoprecipitation assay. (B) The relative affinities of SMAD proteins to the MAN1 mutants. Data are presented as mean ± SEM values from duplicate experiments. *$P < 0.05$ compared to the control by Student's *t*-test. (C) Immunoblotting of full-length mutated FLAG-SMAD1 (SMAD1E-H364Y-F366W) and FLAG-SMAD2 (SMAD2E-Y366A, P377A, W368A, Y366H, W368F and Y366H-W368F) with a combination of full-length wildtype HA-MAN1 after the co-immunoprecipitation assay. (D) The relative affinities of SMAD mutants to the wildtype MAN1 protein. Data are presented as mean ± SEM values from duplicate experiments. *$P < 0.05$ compared to the control by Student's *t*-test. (E-G) Luciferase reporter activation in the cells expressing the indicated MAN1 mutant proteins by TGF-β (E), activin A (F), and BMP4 (G). HepG2 (E, F) and C2C12 (G) cells transiently transfected with the indicated plasmids were treated with each ligand. The cells were lysed, and luciferase assays were performed 72 h post-transfection. Data are presented as mean ± SEM values from duplicate experiments. *$P < 0.05$ compared to the control by Student's *t*-test.

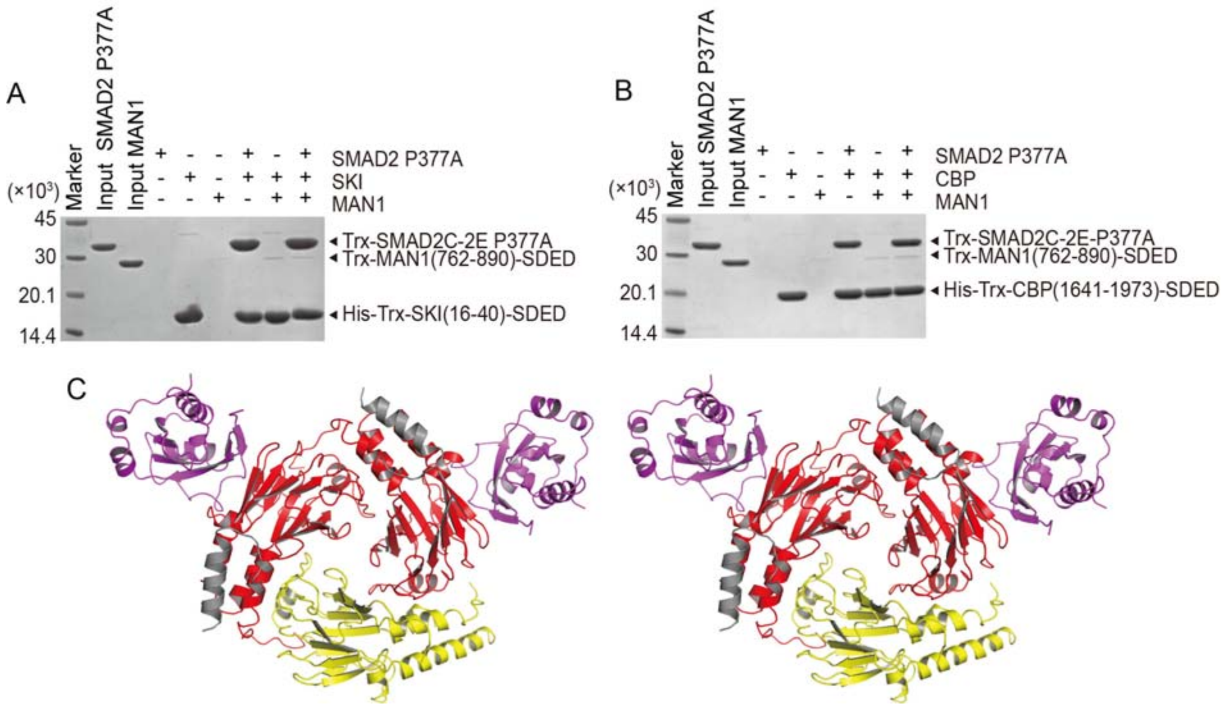

Supplementary Figure 9. Cooperative binding of SMAD cofactors. (A) Interaction of the SMAD2 P377A mutant (Trx-SMAD2C-2E P377A) with MAN1 (Trx-MAN1(762-890)-SDED) and SKI (His-Trx-SKI(16-40)-SDED). (B) Interaction of the SMAD2 P377A mutant (Trx-SMAD2C-2E P377A) with MAN1 (Trx-MAN1(762-890)-SDED) and CBP (His-Trx-CBP(1941-1973)-SDED). After the His-tag pull-down assay, the proteins were separated by SDS-PAGE. The gel images are representative of triplicate experiments. (C) Structural model of the SMAD2 (red)-SMAD4 (yellow)-MAN1 (purple)-SKI (grey) complex (wall-eyed stereo image). The model was prepared using the coordinates of the SMAD2-SMAD4 complex (PDB ID: 1U7V) (44), the SMAD2-SKI complex (PDB ID: 5XOD) (8) and the SMAD2-MAN1 complex.

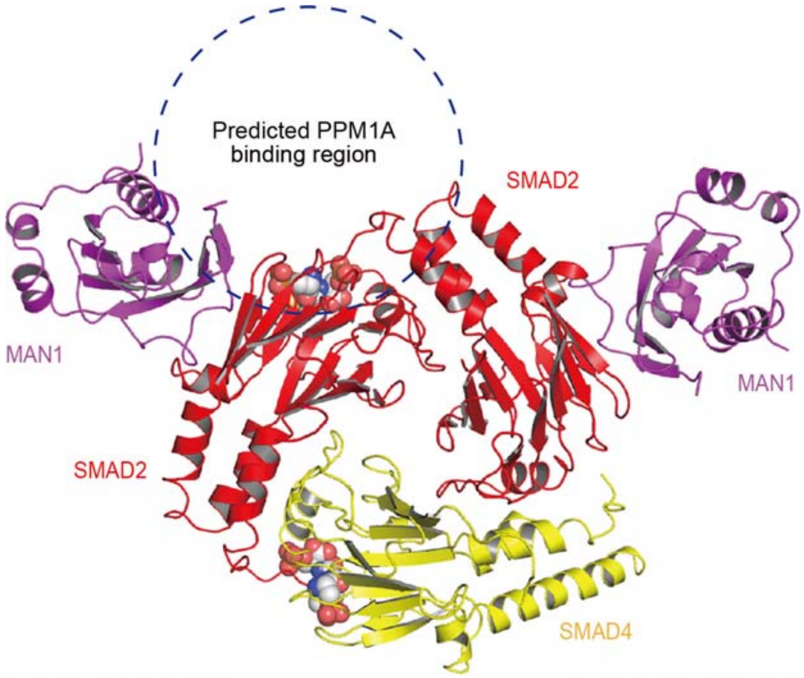

Supplementary Figure 10. R-SMAD-MAN1-PPM1A binding model. The model was prepared using the coordinates of the SMAD2-SMAD4 complex (PDB ID: 1U7V) (44) and the SMAD2-MAN1 complex. SMAD2, SMAD4 and MAN1 are coloured red, yellow and purple, respectively. The phosphorylated SXS motifs of SMAD2 are shown as sphere models. The predicted PPM1A binding site is indicated by a blue dotted circle.
